# A mixed methods evaluation of an integrated adult mental health service model

**DOI:** 10.1186/s12913-019-4501-7

**Published:** 2019-10-14

**Authors:** Diana Beere, Imogen S. Page, Sandra Diminic, Meredith Harris

**Affiliations:** 1 School of Public Health, The University of Queensland, Brisbane, Australia; 2 Policy and Epidemiology Group, Queensland Centre for Mental Health Research, Brisbane, Australia; 3 Queensland Centre for Mental Health Research, Locked Bag 500, Archerfield, QLD 4108 Australia

**Keywords:** Service integration, Mental disorders, Health services, Mixed methods evaluation, Implementation, Outcomes

## Abstract

**Background:**

The Floresco integrated service model was designed to address the fragmentation of community mental health treatment and support services. Floresco was established in Queensland, Australia, by a consortium of non-government organisations that sought to partner with general practitioners (GPs), private mental health providers and public mental health services to operate a ‘one-stop’ mental health service hub.

**Methods:**

We conducted an independent mixed-methods evaluation of client outcomes following engagement with Floresco (outcome evaluation) and factors influencing service integration (process evaluation). The main data sources were: (1) routinely-collected Recovery Assessment Scale — Domains and Stages (RAS–DS) scores at intake and review (*n* = 108); (2) RAS–DS scores, mental health inpatient admissions and emergency department (ED) presentations among clients prospectively assessed at intake and six-month follow-up (*n* = 37); (3) semi-structured interviews with staff from Floresco, consortium partners, private practitioners and the local public mental health service (*n* = 20); and (4) program documentation.

**Results:**

Interviews identified staff commitment, co-location of services, flexibility in problem-solving, and anecdotal evidence of positive client outcomes as important enablers of service integration. Barriers to integration included different organisational practices, difficulties in information-sharing and in attracting and retaining GPs and private practitioners, and systemic constraints on integration with public mental health services. Of 1129 client records, 108 (9.6%) included two RAS–DS measurements, averaging 5 months apart. RAS–DS ‘total recovery’ scores improved significantly (M = 63.3%, SD = 15.6 vs. M = 69.2%, SD = 16.1; *p* < 0.001), as did scores on three of the four RAS–DS domains (‘Looking forward’, *p* < 0.001; ‘Mastering my illness’, *p* < 0.001; and ‘Connecting and belonging’, *p* = 0.001). Corresponding improvements, except in ‘Connecting and belonging’, were seen in the 37 follow-up study participants. Decreases in inpatient admissions (20.9% vs. 7.0%), median length of inpatient stay (8 vs. 3 days), ED presentations (34.8% vs. 6.3%) and median duration of ED visits (187 vs. 147 min) were not statistically significant.

**Conclusions:**

Despite the lack of a control group and small follow-up sample size, Floresco’s integrated service model showed potential to improve client outcomes and reduce burden on the public mental health system. Horizontal integration of non-government and private services was achieved, and meaningful progress made towards integration with public mental health services.

**Electronic supplementary material:**

The online version of this article (10.1186/s12913-019-4501-7) contains supplementary material, which is available to authorized users.

## Background

In late 2014 the Floresco Centre (hereafter, Floresco) commenced operation as an integrated mental health service in Ipswich, Queensland, Australia. We undertook an independent evaluation that examined the effectiveness of Floresco’s service model while also investigating its implementation, including factors enabling or inhibiting service integration and opportunities for improvement. This paper summarises the findings presented in our evaluation report [1].

Floresco was established in response to a problem that emerged in the wake of deinstitutionalisation — the fragmentation of specialised mental health services and non-clinical support services, which has made it difficult for people with mental illness and ongoing functional disability to access the right mix of community-based services at the right time [2–5]. This in turn may lead to poor outcomes for individuals, governments and communities [2–4, 6]. There have been many attempts to develop integrated mental health service models in response to this problem, but relatively few published evaluations [5]. More evidence is needed regarding their implementation and effectiveness, to inform service development and planning.

The need for improved clinical and non-clinical service integration is a recurring theme in mental health plans in many countries [6–10]. In Australia, this need arises because the mental health service system comprises a complex mix of government agencies, private (for-profit) providers, and not-for-profit non-government organisations (NGOs), while responsibility for funding different levels and types of mental health care is dispersed across different tiers of government [3–5]. ‘Service integration’ in this context refers to collaborative attempts by two or more service providers to improve outcomes for individuals with mental illness by breaking down barriers between services (horizontal integration), without necessarily merging into a single organisation (vertical integration) [10]. It may involve one or more of a broad range of strategies — such as formal agreements, joint service planning and provision, single cross-agency care plans, cross-training of staff, shared case records, integrated funding, and service co-location [5] — and can be viewed as a continuum, rather than something that is either achieved or not [11, 12].

Evidence on the effectiveness of models that integrate clinical and non-clinical services is weak, as is the evidence on how best to implement and strengthen integration within complex, multi-sectoral mental health systems. Systematic reviews, of adult/general [5] and youth [12] integration models, and subsequent studies [13], have identified positive clinical and non-clinical outcomes for clients as well as some benefits for services. However, many available studies were retrospectively conducted after the program was implemented, limiting the opportunity for experimental designs; for example, one systematic review found that only 14 out of 40 eligible studies included randomisation procedures in study design [5]. Also the integration mechanisms employed in these initiatives were usually inadequately described; there was little detail about how they worked or what was involved in implementing them [5, 12].

Several evaluations have identified factors that facilitate mental health service integration. These include strong leadership across participating services [5, 10, 14] and co-location [5, 14], which can in turn promote another key enabler, namely effective information-sharing [5, 14, 15]. Further, several studies have emphasised the importance of attending to issues of culture and philosophy, to ensure mutual respect and trust, and shared understandings about the purpose of integration [5, 14, 15].

Barriers have also been considered in some evaluations. For example, a 2009 qualitative evaluation of Jigsaw, an Australian integrated young persons’ mental health program [16], highlighted challenges in bringing together different organisational and professional cultures and maintaining the quality of relationships between partners. The Jigsaw program also had difficulty in recruiting general practitioners (GPs) [16], a problem shared by many Australian *headspace* centres, where workforce issues often present challenges for the ‘seamless’ provision of co-located services for young people [17]. Key challenges for the English Care Programme Approach to integrating care for people with mental illness included patchy implementation, a lack of shared information systems, and care coordinators’ lack of power to exert authority across networks of diverse providers [15]. In the implementation of Integrated Service Networks under the Quebec Mental Health Reform, most barriers related to organisational characteristics such as high staff turnover, concerns about losing autonomy, and leadership problems [10]. Other identified barriers include funding and technology constraints, excessive workloads, ‘turf wars’ among service providers, difficulties in maintaining stakeholder commitment, and barriers to information sharing, including concerns about client confidentiality [5].

## Methods

### Evaluation setting

Floresco was established by a consortium of four NGOs comprising two mental health support providers, a disability support provider, and a tenancy advice and advocacy service. It was intended to operate as a ‘one-stop’ service hub for adults (18 years or older) with mental illness and family members or carers of people with mental illness. The Queensland Government funded the consortium — through the lead agency, Aftercare — to deliver a suite of community-based psychosocial support service types. These services had not previously been available in the area, except to specific groups of people with mental illness (e.g., those being released from correctional facilities). The consortium also established partnerships with the local public mental health service (MHS), GPs, psychology and mental health social work providers, and other community service providers (e.g., drug and alcohol, employment, and housing services) to enable their co-location at Floresco. Further information about Floresco’s structural and operational characteristics is shown in Table 1.

**Table 1:** Structural and operational characteristics of Floresco

Characteristic	Detail
Service area	• Floresco serves residents of Ipswich — a city within the Greater Brisbane metropolitan area, to the west of Brisbane city — and surrounding areas. This service area comprises a mix of urban and rural communities, and includes large areas of relatively high socioeconomic disadvantage [18].
Staffing profile	• Aftercare employed a service manager, part-time clinical team leader, intake officer, and receptionist/administrative assistant. • The three partner NGOs each employed two mental health support workers.
Service types	• Personalised support • Group support • Mutual support and self-help • Family support • Carer support
Payment arrangements	• All Floresco services are delivered free of charge. Psychosocial support services delivered by the consortium NGOs are funded by the Queensland Government; other community support services delivered by co-located NGOs (not part of the consortium) are also directly funded by the Queensland and/or Australian Governments. • Co-located private GPs and allied health practitioners agree, as part of their agreement with the Floresco consortium, not to charge clients for their services. Aftercare bills the Australian Government on their behalf, under the Medicare Benefits Scheme (MBS) and retains a proportion of MBS payments to cover its costs (administrative services, practitioner rooms, etc.). This arrangement is designed to provide an income stream from which to fund the delivery of additional services to those funded by the Queensland and Australian Governments.
Integration mechanisms	• Co-location: all Floresco services are located in the same building • A single triage, intake and assessment process, so that clients have to tell their story only once • A single care plan • Shared client information system, so that all providers involved in an individual’s care can access and update information on that person’s recovery journey • Single practice manual of policies and procedures • Single brand name and logo for use throughout the centre, to minimise confusion for clients • A single outcomes measure — the RAS–DS — for assessing mental health recovery • A single measure of client satisfaction — the Your Experience of Service (YES) questionnaire • Collaborative governance through a Governance Committee comprising representatives from the four consortium NGOs, the local MHS, and several other local community service providers.

Potential clients may be formally- or self-referred. Following an intake assessment, clients may engage in individual and/or group services; progress is reviewed at three-monthly intervals and at discharge. Additional file 1 describes the intended service model, and potential client pathways through Floresco’s services.

We developed a program logic (theory of change) to inform the focus of the evaluation (Additional file 2). It outlines the multiple mechanisms (inputs) required to establish the roles and responsibilities of the consortium NGOs, and the strategies intended to support service integration at Floresco (outputs). The inputs include the memorandum of understanding between Aftercare and its consortium partners, the funding and subcontracting arrangements, the single brand name, and the co-location of other relevant service providers at the centre. The outputs include a single care plan, a shared client information system, a single set of policies and procedures, and standard outcomes and satisfaction measures used throughout Floresco.

### Design

A mixed methods evaluation was conducted between mid-2015 and March 2018. It addressed questions about implementation processes (four questions) and client outcomes (two questions) using five data sources. Table 2 shows the questions and the data sources relevant to each.

**Table 2:** Relationship between the evaluation questions and data sources

		Data sources				
Evaluation questions		A: Observational data and document reviews^a^	B: Stakeholder consultations^b^	C. YES survey^a^	D: Routinely collected client-level data^a^	E: Follow-up study of clients^b^
Process evaluation	Q1. Was an integrated service model implemented at the Floresco Centre as planned?	✓	✓✓	✓		
	Q2. What were the barriers to effective service integration?	✓	✓✓			
	Q3. What were the key enablers of service integration?		✓✓			
	Q4. How could the Floresco service model be improved to achieve better outcomes for clients?		✓✓			
Outcomes evaluation	Q5. Has the Floresco service model improved outcomes for people with mental illness?		✓	✓	✓✓	✓✓
	Q6. To what extent has the service model contributed to improved system outcomes, including reduced use of acute care services?		✓			✓✓

✓✓ Major contribution; ✓ Minor contribution

^a^data source was a routine source

^b^data source was designed for this evaluation

### Data sources

#### Observational data and document reviews

Information about the establishment of Floresco and the development and implementation of its service model was gathered from program documentation (the initial funding proposal, the funding contract and subcontracts, memoranda of understanding, and Floresco’s practice manual), and Governance Committee meetings (meeting papers for the period mid-2014 to March 2018, and observations and notes taken at meetings between mid-2015 and March 2018).

#### Stakeholder consultations

Stakeholder perspectives regarding the extent to which Floresco achieved the goal of mental health service integration were gathered using semi-structured interviews, conducted towards the end of the evaluation period (from August to November 2017). We sought the views of stakeholders involved in a range of aspects of the Floresco service model. Twenty-five potential participants were identified through document reviews and snowball methods, and were invited for interview via email or telephone. Five declined or did not respond. The 20 consenting participants (some holding multiple roles) included Aftercare managers involved in establishing and overseeing Floresco (4), Floresco staff (4), support workers employed by consortium partners (3), consortium partner managers (4), private practitioners (2), MHS staff (5), and Floresco Governance Committee members (10). All received participant information and the question guide (Additional file 3) prior to interview. Interviews (conducted by DB) took 30–60 min, and were recorded and professionally transcribed.

#### YES survey

The Your Experience of Service (YES) is a 35-item client self-report survey developed by the Victorian Department of Health for mental health organisations to gather information about clients’ experiences of care [19] (see Additional file 4). Aftercare engages an independent contractor to conduct the survey annually, and supplied us with the de-identified results for the 34 Floresco clients who completed it in April 2017. These clients comprised 39% of the 87 clients who were targeted for the survey because they were receiving one-to-one support through Floresco at the time. They completed a modified YES instrument in which two original items were replaced with Floresco-specific items, which asked clients to consider all the Floresco services they had used within the previous 3 months and rate (1) ‘how easy you found it to get the right mix of services to help you’ and (2) ‘Floresco’s effectiveness as a “one-stop-shop” for people who want help to improve their mental health’. Responses to all YES items are rated on a six-point scale, ranging either from ‘never’ to ‘always’ or from ‘poor’ to ‘excellent’. The results can be interpreted using individual item-level analysis or summary scores; we chose the former in order to focus only on items that were relevant to the evaluation questions.

#### Routinely collected client-level data

De-identified electronic extracts of routinely collected data were supplied for all referrals received by Floresco between 1 July 2015 to 31 December 2017 (*n* = 2579), of which 83.8% (*n* = 2162) resulted in at least one contact with Floresco. The extracts included demographic characteristics, clinical characteristics, factors affecting mental health (e.g., homelessness, social isolation, history of domestic violence), referral date and source, and scores on the Recovery Assessment Scale — Domains and Stages (RAS–DS), which Floresco clients complete at intake, case review and, where possible, exit. The RAS–DS is a valid and reliable 38-item self-report measure of mental health recovery developed from the Recovery Assessment Scale [20, 21]. Each item is rated on a four-point scale (1 = untrue to 4 = completely true). Scores can be summed to provide a total score and four domain scores: functional recovery (‘Doing things I value’), personal recovery (‘Looking forward’), clinical recovery (‘Mastering my illness’) and social recovery (‘Connecting and belonging’). Percentage scores for each domain are calculated by dividing the total score for that domain by the number of items in that domain and dividing that by 4 (e.g., for ‘Doing things I value’ Score = sum of scores/6/4*100). If there are missing values, the sum of scores is only divided by the number of items that have been rated [22].

#### Follow-up study of clients

A six-month follow-up study of clients was designed to gather information about outcomes and use of acute care services following engagement with Floresco. We included clients referred to Floresco from the local MHS (outpatient and acute inpatient services), because this group is most likely to use acute care services in the short term. The Floresco intake officers determined the eligibility of clients at the intake appointment, and then, with their agreement, introduced them to a researcher (IP or DB) who took them through the informed consent process. Where possible the researcher waited onsite at the time of intake appointments; otherwise, potential participants were contacted by telephone, again with their prior agreement.

**Fig. 1:**
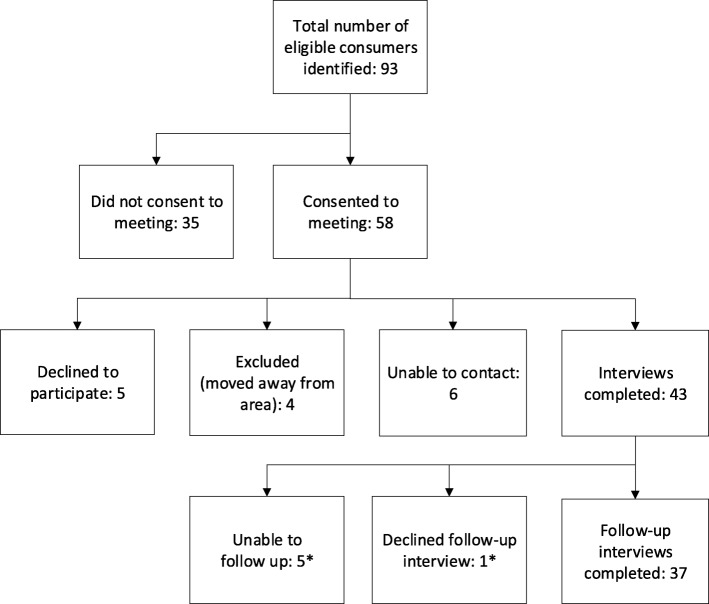
Follow-up study recruitment flowchart. * None of these participants were using Floresco services at the six-month follow-up date

During the recruitment period, October 2016 to September 2017, 93 Floresco clients were identified as eligible for the follow-up study (see Fig. 1). Of these, 43 completed baseline interviews; follow-up interviews were conducted with 37 (86%) approximately 6 months later (mean = 6.3 months; SD: 0.39 months). At follow-up, participants were deemed ‘unable to be contacted’ after three unsuccessful attempts.

Fully-structured baseline and follow-up interviews gathered information about socio-demographic and clinical characteristics, and health service use (e.g., GP and allied health professional consultations) (Additional files 5 and 6). Interviews were conducted in a private space at Floresco or at a library or café convenient to the participant; most took approximately 30 minutes to complete. Responses were recorded directly into the online survey software *Checkbox* via an electronic tablet. Upon completion of each interview, participants received a $20 multi-store gift card and an information sheet listing local MHS and online mental health resources. Interviews were supplemented by extracts of electronic records regarding use of public hospital emergency department (ED) services and acute care beds in the 6 months before and after baseline interview, supplied by the local MHS.

### Data analysis

Constant comparative analysis of qualitative data was undertaken throughout the evaluation (by DB) [23]. Analysis of program documentation and meeting observations informed the question guide and probes for the stakeholder interviews. All transcripts of interviews were checked against the audiofiles to ensure their accuracy and to identify any sections where intonation was significant to the interpretation of the speaker’s meaning. An initial coding framework was developed on the basis of this first review of the data, and in light of the research questions and purpose. This framework was gradually refined with additional codes based on repeated close readings of the transcripts. Coded data were organised to identify themes and sub-themes and to highlight both common and differing perspectives among interview participants.

Quantitative data analyses were conducted (by ISP) using Stata/SE 11.0, using simple descriptive statistics and paired t-tests or their non-parametric equivalent, as required. Tests were two-tailed, with a critical value of *p* < 0.05 indicating statistical significance.

## Results

### Process evaluation

#### Question 1: was an integrated service model implemented as planned?

Stakeholder consultations revealed qualified agreement that an integrated service model had been implemented at Floresco. As one Aftercare manager put it, *It’s a work in progress*. Other informants agreed that, in several ways, the service model was not yet as integrated as intended. In particular, co-location of MHS staff and private allied health providers was inconsistent, most co-located services did not use the shared client information system, it was difficult to recruit and retain GPs, and the mental health support workers employed by each consortium NGO usually did not have the specialist expertise of those organisations (e.g., tenancy advocacy).

**Fig. 2:**
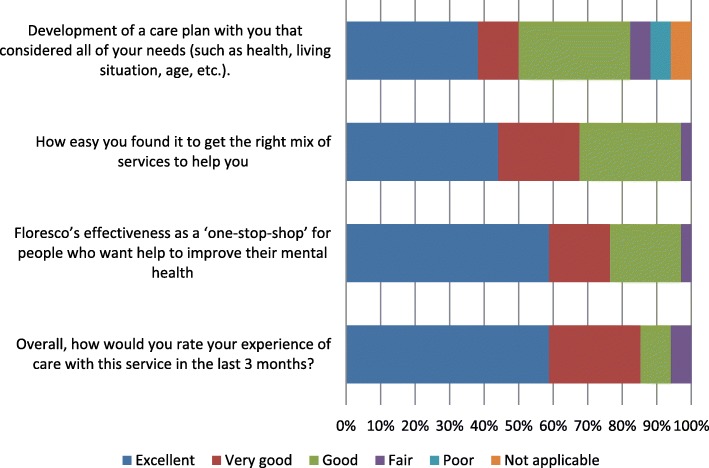
2017 YES survey results — client ratings of Floresco’s performance over the previous 3 months (*n* = 34)

However, from the client’s perspective, Floresco was operating much as the consortium intended. For example, 29 of the 34 YES survey respondents agreed that *Staff worked as a team in your care and treatment (for example, you got consistent information and didn’t have to repeat yourself to different staff)* ‘always’ (27) or ‘usually’ (2). Other relevant YES survey results are shown in Fig. 2. The two Floresco-specific questions received strongly positive responses, and respondents were also positive — albeit less so — about Floresco staff’s development, with them, of care plans that met all of their needs. The full YES survey results for Floresco clients are presented in Additional file 4 (Figures S4.1 and S4.2), together with characteristics of the respondents (Table S4.1).

Stakeholders expressed confidence that Floresco was filling a significant gap in need for psychosocial support and therapeutic group services. They noted that services were in high demand — *100% it has filled a need... that* [can] *be seen by the number of active clients we have and the number of referrals we receive* (Floresco staff member) — and provided support to people not eligible for other services: *we’re a bit like a little basket underneath that can catch the people that don’t qualify for acute services and … long-term services* (Floresco staff member). *There’s a lot of depression and anxiety and domestic violence and crisis situations and emotional crisis and … that’s the stuff that we shouldn’t be dealing with, but the GPs can’t deal with, so it’s really filled that gap* (MHS staff member). Stakeholders also mentioned Floresco’s innovative array of support services — *not just therapeutic work, but things like craft, art, finding your voice, developing a new story, rage, yoga, exercise physiology; also, employment and also just the general support … I think they’ve done a much better job than other agencies* (private practitioner) — and its flexibility in addressing the needs of clients with complex issues: *Usually they can help in some way or they can point you in another direction … they understand that things have to be a little bit more flexible* (MHS staff member)*.*

#### Question 2: what were the barriers to effective service integration?

Together, the stakeholder consultations and the Governance Committee meeting observations and papers revealed that there had been multiple, sometimes inter-related, barriers to the implementation of an integrated service model at Floresco. These included:

- the challenges of bringing staff from four different NGOs together to work as one team
- systemic barriers to integrating with the local MHS
- a range of barriers to systematic information-sharing
- resourcing challenges
- staffing problems
- difficulties in recruiting and retaining GPs and private mental health practitioners
- difficulties in responding to the level of demand as well as the level of clinical need
- complexities related to operating as a consortium
- inconsistent leadership and governance.

Brief explanations of these challenges are included in Table 3, while Additional file 7 provides examples of supporting quotes from stakeholders.

**Table 3:** Barriers to mental health service integration at Floresco

Barriers/challenges	Stakeholders mentioned …
Bringing staff from four different NGOs together to work as one team	• Differences in organisational culture (values, philosophy, tolerance of risk) • Differences in organisational policies, procedures and practices • Having to negotiate over seemingly minor issues • Challenges — for Aftercare, NGO partners, and staff themselves — related to staff supervision
Barriers to integrating with the MHS	• The fact that this initiative was driven by NGOs, rather than mandated by government • Systemic constraints on the MHS’s ability to participate
Barriers to systematic information sharing	• System barriers preventing NGOs from accessing the MHS information system • Practical difficulties preventing MHS staff from using Floresco’s information system • Differing views among the partner NGOs about the importance of record-keeping • Co-located services not using the shared client information system • Difficulty of ensuring that users of the shared client information system enter data correctly and consistently
Resourcing challenges	• Insufficient NGO funding to support necessary components of the service model • Insufficient NGO funding to enable employment of more appropriately qualified, skilled and/or experienced staff to respond to clinical need • Insufficient MHS resources to enable co-location of staff at Floresco
Staffing problems	• High staff turnover, particularly among support workers • Difficulties in recruiting suitable NGO staff, both support workers and managers • Long delays in filling support worker positions • Difficulties maintaining commitment to the integration vision in the context of management changes • Mental health workforce recruitment difficulties in the Ipswich area
Recruiting and retaining GPs and private mental health practitioners	• Constraints on charging a fee • Lack of incentives and support for private practitioners
Responding to demand	• Higher-than-expected demand for services • Insufficient resources to meet demand
Responding to clinical need	• Higher level of clinical need than expected • Inability to meet demand for private practitioners • Insufficient capability among support workers to respond to clinical need
Operating as a consortium	• Additional and more complex staff management problems for Aftercare • Ongoing clinical governance problems • Lack of benefits for clients • Unequal partnerships • Tension between the need to collaborate in the Floresco service model and the pressure to compete in the context of the incoming National Disability Insurance Scheme
Inconsistent leadership and governance	• Over-reliance on the commitment of key personalities • Difficulties maintaining the commitment to collaboration in the face of several management staff changes, particularly within Aftercare • Inconsistent commitment to the Governance Committee among consortium partners • Uncertainty about an appropriate governance model • Lack of strategic focus by the Governance Committee

Not all of the identified barriers had been fully overcome by the time the interviews were conducted, nearly 3 years after Floresco opened: *We were very hopeful and very optimistic that we could overcome a lot of hurdles that have just been too difficult* (MHS staff member). Some optimism remained, however:

*Thinking differently and operating differently will take years … We have to start and work with all of the barriers and all of the difficulties around confidentiality and information sharing and one client information management system … These things all sound easy, but to actually put them into practice is never easy, and it takes time* (Aftercare manager).

#### Question 3: what were the key enablers of service integration?

To the extent that the barriers to integration had been overcome, stakeholders agreed that this was largely due to the strength of the relationships, at management level, between the four consortium NGOs, and between Floresco and the local MHS. These relationships were long-standing in most cases, and were strengthened by the collaborative effort, mutual respect and understanding needed to implement Floresco’s service model. Both Floresco and MHS staff commented on the efforts made by the former to build and maintain strong relationships with the latter, such as Floresco intake staff attending weekly Acute Care Team meetings, which MHS staff recognised as:

*really good of them because sometimes our team meeting is so lengthy and … they're just kind of sitting on the sidelines waiting to speak... they come up every time and it definitely helps because it's really good to have that face-to-face contact and discussion* (MHS staff member).

Other facilitators of service integration included:

- An enabling environment, in which the drive to innovate was supported at senior management and board level.
- Personality factors: the passion and drive of individuals in leadership positions. At the same time, stakeholders acknowledged the risks of relying on one or two key people: *There were key personalities who were so committed to this and wanted it to work and hung in there with it. What’s happened recently is we’ve had a major change of personnel and it’ll be interesting to see what that does to the model* (MHS staff member).
- Committed staff at all levels: *Everyone had hope for this place. Even when there was conflict and a difference of opinion in the way things should be delivered, or managed … they are willing to compromise and to communicate around it... It is the reason we have gotten as far as we have* (Floresco staff member).
- Open communication: *the biggest way to overcome our barriers is to have those conversations and not be scared* (Aftercare manager).
- Co-location of a number of services and supports, all of them accessible via the one reception area, and all operating under the Floresco banner. The latter has helped create a common sense of team identity and purpose among Floresco staff.
- Good reputations and high levels of credibility among the individual consortium NGOs, which have helped achieve buy-in from other organisations as co-located service providers.
- Flexibility, not only in relation to the delivery of services and supports, but also *when the model wasn’t working in one way, the willingness of the steering committee and* [Aftercare] *to turn around and say, right let’s try this or let’s … try something different* (MHS staff member).
- Positive outcomes: as one Aftercare manager explained, the fact that *people have been able to see some outcomes with the clients that they’re working with, or the clients that they’re referring* has helped reinforce their commitment to service integration and to Floresco’s service model as a means of achieving it.

#### Question 4: how could the model be improved to achieve better outcomes for clients?

Improvements to the service model suggested during stakeholder consultations included expansion of the services available, through re-establishment of GP services and increased numbers of co-located private practitioners. Some other suggested improvements — more clinical staff, to better support clients with severe mental illness and complex needs, and an additional intake officer, to reduce the waiting time for intake — would require additional funding.

Several suggestions related to Floresco’s systems and processes. In particular, stakeholders identified the need for a systematic way of sharing information with the local MHS and for improved use of Floresco’s shared client information system. Client information needed to be entered more consistently to allow routine tracking — *making sure that my notes go into the single care plan, that everybody else can see this, that it’s actually building a scaffolding around this individual* (Aftercare manager) — and outcome measures collected and entered more routinely, particularly during case reviews. *The systems weren’t in place to really review people’s recovery plans* (Aftercare manager).

Other suggestions were for ways of managing the difficulties related to operating as a consortium. However, several participants suggested abandoning the subcontracting arrangements between Aftercare and its consortium partners. As one Aftercare manager commented, *A lot of time gets spent on working through challenges. If that money was just with one organisation, you’d just work through it yourself … rather than having to go back and forth*. Another noted that *The bit that’s probably never recognised in consortium models is around how resource intensive running consortia is. If you don’t have an organisation with enough capability to actually do that, invest some of its own dollars, it’s an expensive exercise*.

### Outcomes evaluation

#### Question 5: has the service model improved outcomes for people with mental illness?

Baseline data for the follow-up study participants are presented in Table 4. In most respects, the characteristics of the follow-up study participants were similar to those of the whole Floresco client cohort. However, the mean number of diagnoses per client was higher in the follow-up study group, as were the rate of psychosis, suicide risk and the prevalence of additional factors affecting mental health at intake to Floresco. These differences in the severity and complexity of mental health problems among the follow-up study group were consistent with them having been referred to Floresco from the local MHS, rather than being self-referrals or referrals from GPs or community services.

**Table 4:** Baseline data on Floresco clients

	All Floresco clients who had at least one occasion of service (*n* = 2162)^a^	Follow-up study participants (*n* = 43)
Age: Mean (range)	41 years (18–90)	40 years (19–62)
Sex: % (n)		
Female	60.6% (684)	58.1% (25)
Male	39.4% (445)	41.9% (18)
English as first language: % (n)	–	95.3% (41)
Aboriginal or Torres Strait Islander: % (n)	–	9.3% (4)
Government benefits as main income source: % (n)	–	74.4% (32)
Currently doing any paid work: % (n)	–	23.3% (10)
Highest educational qualification: % (n)		
University qualification (including post-graduate)	–	11.6% (5)
Other post-school qualification	–	62.8% (27)
Year 12 or equivalent	–	7.0% (3)
Year 11 or equivalent	–	2.3% (1)
Year 10 or equivalent	–	9.3% (4)
Year 9 or equivalent	–	7.0% (3)
Referral source: % (n)		
GP or other non-government health care provider	59.1% (872)	0
Self/family/friend	17.1% (252)	0
Other community service	12.1% (179)	0
Public mental health service	11.7% (173)	100% (43)
Mental health diagnosis^bc^		
Mood disorders: % (n)	67.3% (614)	79.1% (34)
Anxiety disorders: % (n)	53.8% (491)	65.1% (28)
Psychosis: % (n)	10.3% (94)	23.3% (10)
Other: % (n)	20.6% (188)	20.9% (9)
Average number of diagnoses per client	1.5	1.9
Suicide risk^c^: % (n)		
Lifetime history of suicide attempt	3.6% (78)	–
Current suicidal ideation within 12 months	3.3% (72)	72.1% (31)
History of self-harm	2.0% (42)	–
Current deliberate self-harm within 12 months	0.7% (15)	30.2% (13)
None of the above	7.1% (153)	–
Additional factors affecting mental health at intake^c^: % (n)		
Social isolation	6.0% (129)	69.8% (30)
Financial strain	–	60.5% (26)
Physical health concern	5.8% (126)	37.2% (16)
History of sexual or physical assault or abuse	2.2% (47)	41.9% (18)
Homeless or at risk of homelessness	1.5% (32)	48.8% (21)
Unemployment/employment issues	–	51.2% (25)
Relationship problems	–	18.6% (8)

^a^ Missing data ranged from 33 to 683 observations

^b^ For the all Floresco clients group, mental health diagnosis was available for 913 clients (42.3%); other diagnosis category includes eating disorders, substance abuse, personality disorders, trichotillomania, sleep disorder, irritability and anger, adult onset ADHD, trauma and stress

^c^ Categories are not mutually exclusive

At baseline, the follow-up study participants reported high rates of suicidal ideation in the previous year (see Table 5). Suicidality was less evident in this group during the 6 months between interviews; however, this finding should be interpreted with caution, given the non-comparability of the timeframes. To the extent that we can assume that use of such services is evenly distributed over time, findings suggest that use of GP and community services remained fairly stable.

**Table 5:** Suicide risk and use of services in the 12 months prior to Floresco intake and the 6 months between initial and follow-up interviews, for follow-up study participants who completed both interviews (*n* = 37)^a^

	12 months prior to Floresco intake	6 months between interviews
Suicide risk: % (n)		
Suicidal ideation	67.6% (25)	32.4% (12)
Self-harm	24.3% (9)	10.8% (4)
Suicide attempt	29.7% (11)	8.11% (3)
None of the above	29.7% (11)	64.9% (24)
GP service use: % (n)		
Saw a GP	100% (37)	97.3% (36)
Median number of consultations (range)	12 (0–50)	4 (0–20)
Median number of consultations for mental health reasons (range)	5 (0–40)	2 (0–15)
Community services use^b^: % (n)		
Employment or financial counselling service	35.1% (13)	4.7% (2)
Emergency/crisis/domestic violence support service	24.3% (9)	18.6% (8)
Alcohol or other drugs service	10.8% (4)	8.1% (3)
Housing/homelessness support service	8.1% (3)	11.6% (5)
Child or family support service	5.4% (2)	10.8% (4)

^a^ As information on the 6 months between interviews was not available for clients who did not complete a follow up interview (*n* = 6), only data on those who completed both interviews were included

^b^ Categories are not mutually exclusive

Table 6 shows RAS–DS results for:

- all Floresco clients who completed the RAS–DS at intake
- all Floresco clients who completed the RAS–DS on two or more occasions
- follow-up study participants who completed both baseline and follow-up interviews.

**Table 6:** RAS–DS results

RAS–DS domain	Mean score (SD)		
	Baseline	Follow-up	Change
All Floresco clients at intake (*n* = 1129)			
Doing things I value	69.6% (16.2)	–	–
Looking forward	64.2% (16.0)	–	–
Mastering my illness	58.6% (16.7)	–	–
Connecting and belonging	66.4% (17.5)	–	–
Overall	64.7% (14.0)	–	–
All Floresco clients who completed the RAS–DS on two (or more) occasions (*n* = 108)^a^			
Doing things I value	70.4% (18.3)	72.4% (17.3)	+ 2.0% (15.3)
Looking forward	62.9% (16.3)	69.9% (17.9)	+ 7.0% (16.8)***
Mastering my illness	56.1% (17.7)	65.6% (18.6)	+ 9.5% (18.9)***
Connecting and belonging	63.8% (18.4)	68.8% (18.4)	+ 5.0% (15.6)**
Overall	63.3% (15.6)	69.2% (16.1)	+ 5.9% (14.3)***
Follow-up study participants who completed both baseline and follow-up interviews (*n* = 37)^b^			
Doing things I value	72.0% (14.8)	76.6% (14.9)	+ 4.6% (20.5)
Looking forward	65.6% (15.0)	73.0% (14.3)	+ 7.4% (21.6)*
Mastering my illness	57.9% (17.1)	71.2% (14.4)	+ 13.3% (25.9)**
Connecting and belonging	66.2% (17.5)	74.2% (17.3)	+ 8.0% (25.4)
Overall	65.4% (13.1)	73.8% (13.4)	+ 8.3% (20.4)*

Note: higher scores indicate higher levels of recovery

* *p* < 0.05; **p < 0.01; ***p < 0.001

^a^ Average period between baseline and follow-up was 5.1 months. Dates when the RAS–DS was completed were available for 78 of the 108 clients (72.2%)

^b^ Average period between baseline and follow-up was 6.3 months

Baseline results for all three groups were very similar: mean overall scores ranged from 63.3 to 65.4% and differences in individual measures between groups ranged from 2.1 to 2.7%.

Among Floresco clients who completed the RAS–DS on two or more occasions, significant increases in self-reported mental health recovery were seen across three of four domains (all but ‘Doing things I value’) and there was a significant difference between overall baseline (M = 63.3%, SD = 15.6) and follow-up (M = 69.2%, SD = 16.1) RAS–DS results (t(107) = 4.25, *p* < 0.001) (Table 6, middle section). Similar results were seen for the 37 follow-up study participants: RAS–DS ‘total recovery’ scores improved between baseline (M = 65.4%, SD = 13.1) and six-month follow-up (M = 73.8%, SD = 13.4) (t(36) = 2.48, *p* = 0.018) (Table 6, lower section). So did scores for RAS–DS domains of ‘Looking forward’ (*p* = 0.043) and ‘Mastering my illness’ (*p* = 0.004), but not ‘Doing things I value’ (*p* = 0.184) or ‘Connecting and belonging’ (*p* = 0.063).

Several participants in the stakeholder consultations expressed the view that the Floresco service model was contributing to improved mental health outcomes. For example, one of the private practitioners commented that they could:

*see* *the outcomes in clients I've tracked*. *Even in the sense that we get referred, highly suicidal ones who won’t go to hospital … Because of the support and the clinical work we’ve done here, we’ve been able to, I guess, keep them alive.*

**Fig. 3:**
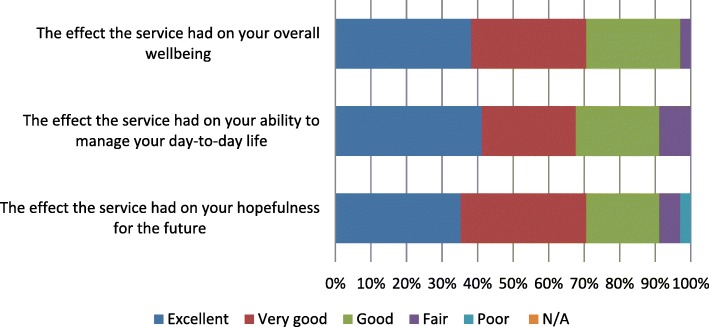
2017 YES survey results — client ratings of Floresco’s effectiveness in improving their mental health and wellbeing (*n* = 34)

The 34 Floresco clients who participated in the YES survey reported positively on the differences that Floresco had made in their lives. The survey asked about the effects that Floresco had on their overall wellbeing, their ability to manage their day-to-day lives, and their hopefulness for the future; as shown in Fig. 3, in all cases about 70% of respondents rated these effects as either excellent or very good. A large majority also rated their overall experience of service at Floresco as either excellent (59%) or very good (26%), implying that they were satisfied with the outcomes.

#### Question 6: to what extent has the service model contributed to improved system outcomes?

Use of hospital ED services and acute care beds by follow-up study participants in the 6 months pre- and post-intake to Floresco can be seen in Table 7. Decreases in hospital admissions and ED attendance were recorded, along with decreases in the median length of stay. The total number of occupied bed days for the cohort declined from 129 to 21, while the total duration of ED attendances declined by more than 50%. However, only a few of the participants were admitted to hospital or attended ED. Likely due to the small sample size, the decline in the number of participants admitted to hospital post-intake was not statistically significant according to McNemar’s test (chi2(1) = 4.57; *p* = 0.057); nor was the decline in the number of participants attending ED post-intake (chi2(1) = 4.50; *p* = 0.070).

**Table 7:** Use of public hospital ED and inpatient services by follow-up study participants (*n* = 43) pre- and post-intake to Floresco

Public hospital service use	6 months prior to Floresco intake	6 months between interviews
Hospital admission:		
Admitted for mental health reasons: % (n)	20.9% (9)	7.0% (3)
Total number of admissions for cohort (n)	12	10
Total number of occupied bed days for cohort (n)	129	21
Median number of admissions (range)	1 (1–3)	1 (1–8)
Median length of stay per admission in days (range)	8 (1–46)	3 (1–17)
Emergency department attendance:		
Attended for mental health reasons: % (n)	34.8% (15)	6.3% (7)
Total number of attendances for cohort (n)	34	20
Total duration of attendances for cohort, in minutes	2834	1211
Median number of attendances (range)	1 (1–6)	2 (1–7)
Median length of stay per attendance, in minutes (range)	187 (50–360)	147 (47–320)

Despite the small sample size, the findings in Table 7 lend some support to the belief among stakeholders that the Floresco service model has the potential to lead to improved system outcomes, in terms of more effective use of scarce hospital and MHS resources. For example, one MHS staff member commented that they knew of several Floresco clients who, based on past experience:

*would have presented* [to ED] *so many more times for assessments because ‘there is something but I don't know what I need’. Just needing to talk to a professional, but they didn't necessarily need hospitalisation*.

Another MHS staff member referred to Floresco’s contribution to easing the pressure on the Acute Care and Continuing Care teams:

*it's taken a lot of pressure off us to actually really just focus on managing crisis, managing acuity and doing that, and then, once we can* *stabilise it … maybe people that might have gone to case management, they don't need to go there, they can go to Floresco and get some follow-up*.

## Discussion

In an area with apparently high levels of previously unmet need for mental health and psychosocial support services, Floresco has been a valuable addition to the mental health service system — the more so because its service model provides clients with seamless access to a wide range of services.

While it is still a work in progress, we found that horizontal service integration between multiple NGO service providers and private mental health practitioners has largely been achieved at Floresco, as evidenced by their co-location, their implementation of a shared client information system, and their adoption of a single set of processes, outcome measures and practice standards. However, despite the development of a strong collaborative relationship between Floresco and the local MHS, intersectoral integration has not yet been achieved. This is due mainly to public health system barriers that currently prevent NGOs from accessing client data via the MHS information system, together with resource constraints that limit the possibilities for MHS staff to co-locate at Floresco and use its shared client information system.

As found in previous evaluation studies [5, 10, 14], committed leadership and strong relationships were key enablers of service integration at Floresco; in this case they have been particularly important because the drive to innovate has come from outside government — that is, from the funded NGOs, rather than the funding body. That said, much of the leadership has been provided by a few passionate individuals whose departures from key positions have exacerbated the challenge of maintaining a shared vision and understanding of Floresco’s integrated service model across the four consortium partners.

Another important enabler of service integration identified in previous studies [5, 14], co-location of services, has been combined in Floresco’s case with the adoption of a single brand name; this has helped to develop a strong sense of team identity at Floresco, despite staff being employed by four different NGOs. While co-location of MHS staff at Floresco has not yet been possible in an ongoing way, Floresco staff have nevertheless gained some of the benefits of co-location by regularly attending the Acute Care Team’s weekly meetings. This frequent face-to-face contact over 3 years has helped to build mutual respect and understanding, and contributed to effective information-sharing between Floresco and the MHS, even in the absence of a shared information system.

Several of the significant challenges involved in implementing an integrated service model at Floresco arose as a result of the consortium approach, which complicated issues in relation to clinical governance and the recruitment and management of support workers, and may have contributed to high staff turnover and lengthy delays in filling vacant positions. These problems added to the challenge of bringing different organisational cultures together, which were noted in the evaluation of the Jigsaw program [16]. Like Jigsaw and many *headspace* centres, Floresco has had difficulty recruiting and retaining GPs [16, 17]; it has also had limited success in recruiting MBS-funded private mental health practitioners. As a result, Floresco has not only struggled to meet the high level of clinical needs among its clients, but has also been unable to secure a supplementary funding stream that might have made it possible to recruit and retain an additional intake officer and/or better qualified support staff, and thus reduce the length of its waiting list.

Barriers to service integration identified in other studies but not at Floresco include difficulties maintaining stakeholder commitment [5] and concerns about losing autonomy [10]. Maintaining stakeholder commitment may have been easier in Floresco’s case because of the leadership provided by key personalities, particularly in the first 2 years, and because of the strong relationships that already existed between the consortium NGOs and the MHS prior to Floresco’s establishment. Another factor that mitigated the difficulties of maintaining stakeholders’ commitment to service integration was their perception that Floresco’s service model was contributing to positive mental health outcomes for clients.

None of the key informants mentioned any concerns about losing autonomy, but such concerns may have contributed to some of the challenges involved in bringing staff from four NGOs together to work as one team; certainly it seems that Aftercare’s NGO partners had some concerns about losing control of the staff they placed at Floresco. However, because their involvement in Floresco was limited to only a few staff in each case — it did not involve the whole organisation or impact on its culture or systems — the three partner NGOs may never have perceived any threats to their autonomy.

‘Turf wars’ among service providers, which have caused problems for other mental health service integration initiatives [5], were also not evident at Floresco. However, there were some signs that they might emerge following the implementation of Australia’s National Disability Insurance Scheme, under which collaborative partnerships such as those in operation at Floresco could be undermined by the pressure to compete.

We found numerous opportunities for improvements to the Floresco service model that may help to achieve even better mental health outcomes for clients. In particular, the model has been inadequately funded to meet the higher-than-expected clinical mental health needs of the local population and to cover the costs involved in holding its components together. That said, the current level of funding could be used more flexibly, and potentially more effectively, if Aftercare were not locked into subcontracting arrangements with its consortium partners. These arrangements limit the possibilities for employing a more appropriate mix of clinical staff and mental health support workers. Moreover, if the consortium NGOs were to provide their services in kind, rather than via subcontracted outputs, they would be better placed to deliver services in their speciality areas, and the potential benefits to clients of bringing together a group of NGOs with different areas of expertise could be realised. Abandoning the subcontracting arrangements would also allow Floresco staff to focus more on improving systems and processes, rather than on managing overly complex staffing arrangements and negotiating compromises among the consortium NGOs over clinical governance issues. In particular, it seems likely that clients’ mental health outcomes could be improved if Floresco staff and co-located providers could be trained and supported to make better use of the shared client information system.

Despite the considerable challenges to integration identified in this study, Floresco’s service model appears to have contributed to positive mental health outcomes among clients who have significant mental health and functional difficulties but do not require inpatient care and may not meet the eligibility threshold for continuing community-based care through the MHS. Our study found that clients appreciated the way Floresco operated as a one-stop shop for a variety of mental health services and supports, were satisfied with the services they received, and improved in recovery during their engagement with Floresco. While clients referred by the MHS reduced their number of ED presentations and hospital admissions following their engagement with Floresco, the changes were not statistically significant. They may nevertheless have positive resource implications for public sector acute and continuing care mental health services, because the *p* values were trending towards significance; another study with a greater sample size would be required to assist in determining whether significant reductions in acute mental health services are achievable from this model.

### Limitations of the study

This study had several limitations. The process component might have benefitted from multiple interviews with each informant, at different points in the implementation of the service model. Analysis of the qualitative data by two or more members of the research team might also have enhanced the perceived quality of the process component, although it would not necessarily have led to different findings [24]. Either way, resource constraints limited our quality controls for this component of the study to triangulation (multiple sources of data), the use of a professional transcription service, and verification of the transcripts. Researcher bias was not an issue, given that this was an independent study.

A control group might have enabled us to more conclusively attribute the significant self-reported mental health recovery experienced by Floresco clients (as measured by the RAS–DS) to the Floresco service model. Our research design, as originally planned, included a comparison group recruited from another geographical region, but recruitment of sufficient participants for this proved not to be feasible within the available timeframe.

Other limitations related to the completeness of the routinely collected data. Improving this would require all users of the shared client information system — including not only Floresco staff, but also GPs, private practitioners and staff of co-located services — to be trained to enter data in a consistent manner, and to have shared understandings of the multiple benefits of collecting high quality client data. Matched RAS–DS scores were available for only 9.6% of Floresco clients who completed a RAS–DS at intake; however this is consistent with other routine collections. For example, for the evaluations of Australia’s Access to Allied Psychological Services and Partners in Recovery programs, overall outcomes data were available for only 13.0% and 12.8% of the program’s clients respectively [25, 26].

## Conclusions

This evaluation shows that implementing a new integrated model is challenging in the Australian mental health service environment, where there are so many different funders and providers. However, it also shows that horizontal service integration of NGO and private practitioner services with public MHS is potentially achievable. Key success factors are an enabling environment, committed and consistent leadership, a high level of stakeholder buy-in, strong relationships characterised by open communication, and adequate funding. Findings indicate that Floresco clients improved in recovery during their engagement with the centre. However, more robust study designs are needed to determine whether these improvements are attributable to Floresco’s integrated service model.

## Additional files

Additional file 1: Planned Floresco service model and client pathways. (DOCX 172 kb)

Additional file 2: Floresco program logic diagram. (DOCX 203 kb)

Additional file 3: Floresco service model evaluation: Questions for semi-structured stakeholder interviews. (DOCX 14 kb)

Additional file 4: 2017 YES survey results for Floresco clients. (DOCX 32 kb)

Additional file 5: Baseline interview guide. (DOCX 80 kb)

Additional file 6: Follow-up interview guide. (DOCX 67 kb)

Additional file 7: Examples of stakeholder comments on barriers to service integration at Floresco. (DOCX 39 kb)

## Data Availability

The datasets generated and/or analysed during this study cannot be made publicly available, due to restrictions on their use as well as the ethically sensitive nature of the research. The study participants did not consent to the sharing of their data. Further information about the data is available from the corresponding author.

## References

[1] Beere D, Page IS, Diminic S, Harris M (2018). Floresco Centre service model evaluation: Final report.

[2] National Mental Health Commission (2014). Contributing lives, thriving communities: Report of the National Review of Mental Health Programmes and Services.

[3] Rosenberg S, Hickie I. Managing madness: mental health and complexity in public policy. Evidence Base. 2013(3):1–19.

[4] The Economist Intelligence Unit. Mental health and integration. Provision for supporting people with mental illness: a comparison of 15 Asia Pacific countries. London: The Economist Intelligence Unit Limited; 2016.

[5] Whiteford H, McKeon G, Harris M, Diminic S, Siskind D, Scheurer R (2014). System-level intersectoral linkages between the mental health and non-clinical support sectors: a qualitative systematic review. Aust NZ J Psychiat.

[6] Ellis LA, Churruca K, Braithwaite J (2017). Mental health services conceptualised as complex adaptive systems: what can be learned?. Int J Ment Health Sy.

[7] Department of Health (2017). The fifth national mental health and suicide prevention plan.

[8] Queensland Health (2016). Connecting care to recovery 2016–2021: a plan for Queensland's state-funded mental health, alcohol and other drug services.

[9] Queensland Mental Health Commission. Queensland mental health, drug and alcohol strategic plan 2014–2019. Brisbane: Queensland Government; 2014.

[10] Fleury M-J, Grenier G, Vallée C, Aubé D, Farand L. Implementation of Integrated Service Networks under the Quebec Mental Health Reform: facilitators and barriers associated with different territorial profiles. Int J Integr Care. 2017;17(1):3. 10.5334/ijic.2482 PMC5630082 29042845

[11] Heyeres M, McCalman J, Tsey K, Kinchin I. The complexity of health service integration: a review of reviews. FrontPublic Health. 2016;4:223. 10.3389/fpubh.2016.00223 PMC5066319 27800474

[12] Hetrick SE, Bailey AP, Smith KE, Malla A, Mathias S, Singh SP, O'Reilly A, Verma SK, Benoit L, Fleming TM (2017). Integrated (one-stop shop) youth health care: best available evidence and future directions. Med J Aust.

[13] Dunt DR, Benoy AW, Phillipou A, Collister LL, Crowther EM, Freidin J, Castle DJ. Evaluation of an integrated housing and recovery model for people with severe and persistent mental illnesses: the Doorway program. Aust health review: a publication of the Australian Hospital Association. 2017;41(5):573–81. 10.1071/AH16055 29224600

[14] Ion A, Sunderji N, Jansz G, Ghavam-Rassoul A. Understanding integrated mental health care in “real-world” primary care settings: what matters to health care providers and clients for evaluation and improvement? Fam Syst Health. 2017;35(3):271–82. 10.1037/fsh0000294 28805405

[15] Goodwin N, Lawton-Smith S. Integrating care for people with mental illness: the Care Programme Approach in England and its implications for long-term conditions management. Int J of Integr Care. 2010;10:e040. 10.5334/ijic.516 PMC4098094 25035692

[16] Callaly T, Dodd S, Ackerly C, Hantz P, Oshea M, Berk M. Description and qualitative evaluation of Jigsaw, an integrated young persons mental health program. Australas Psychiatry. 2009;17(6):480–3. 10.1080/10398560902957119 20001371

[17] Hilferty F, Cassells R, Muir K, Duncan A, Christensen D, Mitrou F, Gao G, Mavisakalyan A, Hafekost K, Tarvedi Y, et al. Is *headspace* making a difference to young people's lives? Final report of the independent evaluation of the *headspace* program. Sydney: Social Policy Research Centre, UNSW; 2015.

[18] Australian Bureau of Statistics (2018). 2033.0.55.001 - Census of Population and Housing: Socio-Economic Indexes for Areas (SEIFA), Australia, 2016.

[19] Australian Institute of Health and Welfare. “Your Experience of Service survey instrument”; 2018. Retrieved 15 March, 2018, from https://www.aihw.gov.au/reports/mental-health-services/mental-health-services-in-australia/national-mental-health-committees/mental-health-information-strategy-standing-commit/your-experience-of-service-survey-instrutment.

[20] Hancock N, Scanlan JN, Honey A, Bunday AC, O'Shea K. Recovery Assessment Scale – Domains and Stages (RAS-DS): its feasibility and outcome measurement capacity. Aust NZ J Psychiat. 2014;49(7):624–33. 10.1177/0004867414564084 PMC4941096 25526940

[21] Scanlan JN, Hancock N, Honey A. The Recovery Assessment Scale – Domains and Stages (RAS-DS): sensitivity to change over time and convergent validity with level of unmet need. Psychiatry Res. 2018;261:560–4. 10.1016/j.psychres.2018.01.042 29407723

[22] Hancock N, Scanlan JN, Bundy AC, Honey A. Recovery Assessment Scale - Domains & Stages (RAS-DS) manual - version 2. Sydney: University of Sydney; 2016.

[23] Thorne S (2000). Data analysis in qualitative research. Evid Based Nurs.

[24] Cohen D, Crabtree B. Evaluative criteria for qualitative research in health care: controversies and recommendations. Ann Fam Med. 2008;6(4):331–9. 10.1370/afm.818 PMC2478498 18626033

[25] Bassilios B, Nicholas A, Reifels L, King K, Fletcher J, Machlin A, Ftanou M, Blashki G, Burgess P, Pirkis J. Achievements of the Australian Access to Allied Psychological Services (ATAPS) program: summarising (almost) a decade of key evaluation data. Int J Ment Health Syst. 2016;10:61. 10.1186/s13033-016-0092-4 PMC5037884 27708698

[26] Hancock N, Scanlan JN, Gillespie JA, Smith-Merry J, Yen I (2017). Partners in Recovery program evaluation: changes in unmet needs and recovery. Aust health rev : a publication of the Australian Hospital Association.

