# Supplementary figures and images for: A mixed methods evaluation of an integrated adult mental health service model
Source: BMC Health Serv Res. 2019 Oct 14;19:691. doi: 10.1186/s12913-019-4501-7 (PMC6791005; doi:10.1186/s12913-019-4501-7)

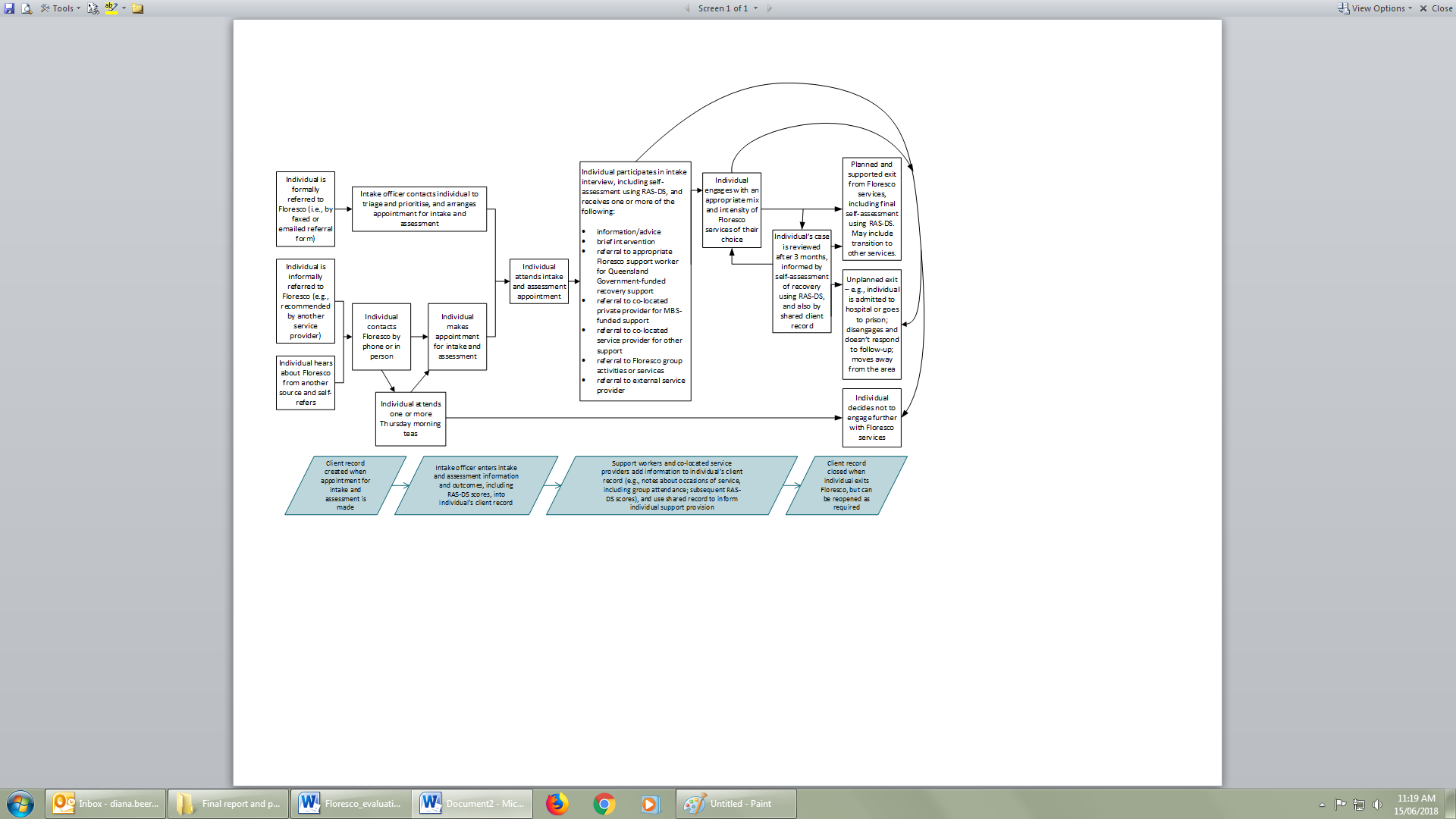


**Additional file 1: Planned Floresco service model and client pathways**

Supplement: Supplementary file 1 — Planned Floresco service model and client pathways. (DOCX 172 kb) [file 12913_2019_4501_MOESM1_ESM.docx]

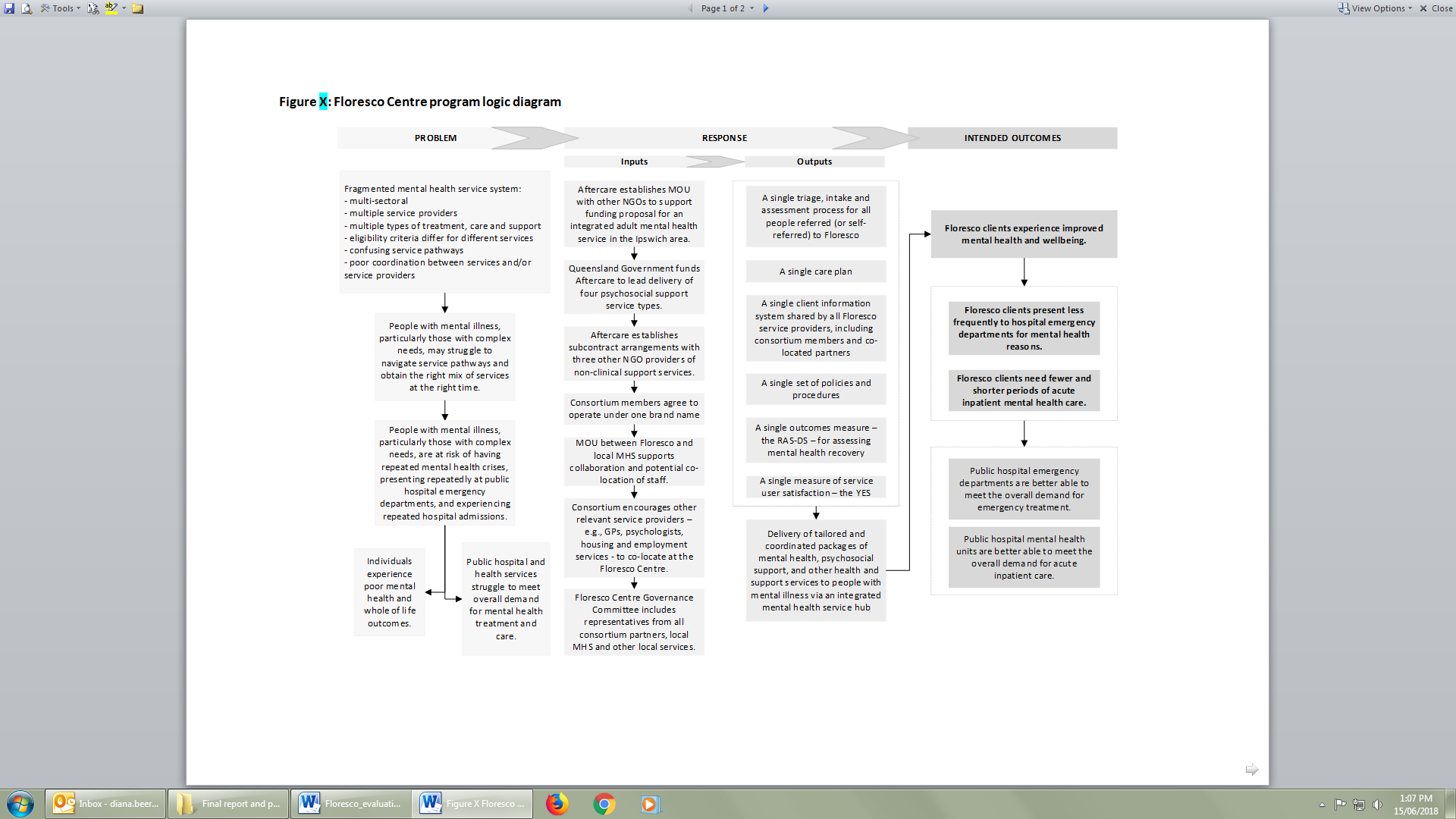


**Additional file 2: Floresco program logic diagram**

Supplement: Supplementary file 2 — Floresco program logic diagram. (DOCX 203 kb) [file 12913_2019_4501_MOESM2_ESM.docx]
