# Supplementary material for: A mixed methods evaluation of an integrated adult mental health service model
Source: BMC Health Serv Res. 2019 Oct 14;19:691. doi: 10.1186/s12913-019-4501-7 (PMC6791005; doi:10.1186/s12913-019-4501-7)
Supplement: Supplementary file 3 — Floresco service model evaluation: Questions for semi-structured stakeholder interviews. (DOCX 14 kb) [file 12913_2019_4501_MOESM3_ESM.docx]

**Additional file 3: Floresco service model evaluation: Questions for semi-structured stakeholder interviews**

**Standard questions**

What is (or was) your connection to the Floresco Centre?

For how long have you been (or were you) connected with the Floresco Centre?

How would you rate your knowledge about Queensland's mental health service system? (e.g., on a scale of 1 to 10, with 1 being 'very limited' and 10 being 'very comprehensive')

A common criticism about Queensland's mental health system is that better service integration is needed. What does 'better service integration' mean to you?

What is your understanding of the motivations behind the establishment of the Floresco Centre?

To the best of your knowledge, has the integrated mental health service model been implemented at the Floresco Centre as planned?

Does it operate as intended?

Is it consistent with what was envisaged?

If not, in what ways does it differ?

To what extent, in your view, has the Floresco Centre filled a need for mental health consumers? Can you explain the basis for that judgement?

What have been the main barriers, from your perspective, to implementing an integrated service model at the Floresco Centre?

Have these barriers been overcome, and if so, how has this been achieved?

To the extent that service integration has been achieved at the Floresco Centre, what do you think have been the key factors that have enabled this?

In your view, is Floresco’s integrated service model sustainable over time? Why, or why not?

How, in your opinion, could the Floresco service model be improved to achieve better mental health and whole-of-life outcomes for consumers?

Based on what you know of the history of Floresco's development, as well as how it operates now, what do you think are the key learnings about service integration?

Thinking particularly about the aim of improving service integration, is there anything that we haven’t already discussed that you would suggest should be done differently next time?

Other than what we’ve already discussed, is there any advice that you would give to other organisations wanting to establish an integrated mental health service?
