# Supplementary material for: A mixed methods evaluation of an integrated adult mental health service model
Source: BMC Health Serv Res. 2019 Oct 14;19:691. doi: 10.1186/s12913-019-4501-7 (PMC6791005; doi:10.1186/s12913-019-4501-7)
Supplement: Supplementary file 4 — 2017 YES survey results for Floresco clients. (DOCX 32 kb) [file 12913_2019_4501_MOESM4_ESM.docx]

**Additional file 4: 2017 YES survey results for Floresco clients**

Aftercare conducts an annual survey about clients’ experience of service across all of their service sites, using the Your Experience of Service (YES) survey instrument. The standard version of this instrument comprises 35 items within four categories: experience, outcomes, open-ended and demographics. The 17 ‘experience’ items ask how often a person has experienced specific service characteristics over the past three months, and are rated on a scale of 1 = Never to 5 = Always. Nine ‘outcomes’ items ask clients to rate aspects of the service’s performance over the previous three months on a scale of 1 = Poor to 5 = Excellent. The remainder of the standard instrument comprises two open-ended and seven demographic items.^^[[1]](#footnote-1)^^

Aftercare’s 2017 survey targeted clients of its clinical, community and residential services across Queensland and New South Wales. It involved 645 telephone interviews with trained interviewers. A total of 34 Floresco clients participated; they comprised 39 per cent of the 87 Floresco clients who were contacted and invited to participate in the survey because they were receiving one-to-one support from a mental health support worker at the time. These clients responded to a Floresco-specific version of the YES, which deletes two ‘experience’ items that are not relevant to Floresco, but includes two additional ‘outcomes’ items. These items ask respondents to use the same 1–5 (Poor to Excellent) scale to rate:

- Floresco’s effectiveness as a ‘one-stop shop’ for people who want help to improve their mental health
- how easy they found it to get the right mix of services to help them.

Of the 34 respondents to the Floresco-specific version of the YES, 24 were female, 4 were of Aboriginal and/or Torres Strait Islander origin, and all reported the main language spoken at home as English. Table 4.1 shows the respondents’ demographic characteristics, together with those of the 1129 Floresco clients whose records were analysed for the Floresco service model evaluation.

**Table 4.1: Demographic characteristics of Floresco clients (n=1129) and the subset of clients who responded to Aftercare’s 2017 YES survey (n=34)**

| **Characteristic** | | **All Floresco clients (n=1129)** | **Floresco YES survey respondents (n=34)** |
| --- | --- | --- | --- |
| Gender | |  |  |
|  | Female | 60.6% (684) | 70.6% (24) |
|  | Male | 39.4% (445) | 29.4% (10) |
| Main language spoken at home | |  |  |
|  | English | - | 100.0% (34) |
| Are you of Aboriginal or Torres Strait Islander origin? | |  |  |
|  | Yes | - | 11.8% (4) |
|  | No | - | 85.3% (29) |
|  | Declined | - | 2.9% (1) |
| Age group  18-24  25-34  35-44  45-54  55-64  65+ | | 10.1% (114)  25.2% (285)  26.7% (301)  20.5% (231)  14.4% (163)  3.1% (35) | -  23.5% (8)  23.5% (8)  35.3% (12)  17.6% (6)  - |
| How long have you been receiving care from this service on this occasion? | |  |  |
|  | Less than 24 hours | - | 0.0% (0) |
|  | 1 day - 2 wks | - | 5.9% (2) |
|  | 3-4 weeks | - | 2.9% (1) |
|  | 1-3 months | - | 55.9% (19) |
|  | 4-6 months | - | 17.6% (6) |
|  | More than 6 months | - | 17.6% (6) |

Overall, responses to the Floresco-specific version of the YES survey were very positive, with a majority of the 34 respondents rating their overall experience of service at Floresco as either excellent (59%) or very good (26%). Figure 4.1 presents the results of the ‘experience’ items, while Figure 4.2 shows those for the ‘outcomes’ items.

**Figure 4.1: Results of ‘experience’ items** **of Aftercare’s 2017 YES survey for Floresco clients**

**Figure 4.2: Results of ‘outcomes’ items of Aftercare’s 2017 YES survey for Floresco clients**

1. For more information about the YES survey instrument, see <https://www.aihw.gov.au/reports/mental-health-services/mental-health-services-in-australia/national-mental-health-committees/mental-health-information-strategy-standing-commit/your-experience-of-service-survey-instrutment> [↑](#footnote-ref-1)
