# Supplementary material for: A mixed methods evaluation of an integrated adult mental health service model
Source: BMC Health Serv Res. 2019 Oct 14;19:691. doi: 10.1186/s12913-019-4501-7 (PMC6791005; doi:10.1186/s12913-019-4501-7)
Supplement: Supplementary file 5 — Baseline interview guide. (DOCX 80 kb) [file 12913_2019_4501_MOESM5_ESM.docx]

**Additional file 5: Baseline interview guide**

| Floresco Evaluation |
| --- |
| Paper interview reference guide: Baseline interviews  Notes:   - If there is no arrow next to an answer then go to the next question - If there is an arrow next to an answer then go to the question indicated - If the question does not say “multiple responses possible” then only one response should be recorded |

**1. Today's date _____________________**

**2. Interview number**

- Floresco 1
- Floresco 2

**To ensure your responses remain confidential, I'm only going to record an ID code for you, rather than your name.**

**<ID code is a combination of numbers, letters from the participant’s name and their date of birth>**

**3. Participant ID code ________________**

**»» I'd like to start by asking for some basic demographic information about you, please.**

**4. What is your age, in years? ____________**

If declined, use 99.

**5. What gender do you identify as: male, female, or other? Or would you prefer not to say?**


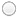
 Male
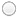
 Other


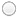
 Female
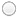
 Prefer not to say

**6. Are you of Aboriginal or Torres Strait Islander origin?**

- No
- Yes, Aboriginal
- Yes, Torres Strait Islander
- Yes, both Aboriginal & Torres Strait Islander
- Declined

**7. What is your first language?**


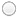
 English
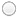
 Declined


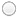
 Other: ____________

**8. What is the highest year of primary or secondary school you completed?**

- Year 12 or equivalent
- Year 11 or equivalent
- Year 10 or equivalent
- Year 9 or equivalent
- Year 8 or below
- Never attended school
- Declined

**9. Have you completed a trade certificate, diploma, degree or any other educational qualification?**


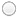
 Yes -> go to Q11


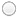
 No -> go to Q12


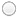
 Declined -> go to Q12

**10. What is the highest level qualification you have completed?**

- Trade certificate or apprenticeship
- Technician's cert./Advanced cert.
- TAFE certification
- Associate diploma
- Undergraduate diploma
- Bachelor degree
- Postgraduate qualification
- Declined

**11. What is currently your main source of income?**

For this question 'currently' means now and over the last 2‐4 weeks.

- Salary or wages -> go to Q13
- Self-employed -> go to Q13
- Government benefits -> go to Q12
- Superannuation/investments -> go to Q13
- No income -> go to Q13
- Declined -> go to Q13
- Other: ____________-> go to Q13

**12. What is the main type of government benefit you're currently receiving?**

i.e. the benefit that currently provides the largest amount of regular income

- ABSTUDY or AUSTUDY
- Carer payment
- Disability support pension
- Newstart
- Parenting payment
- Sickness allowance
- Special benefit
- Declined
- Other: ______________

**»» Now I have a few questions about your physical and mental health.**

**13. In general, thinking about the last 6 months, would you describe your physical health as excellent, very good, good, fair or poor?**

- Excellent
- Very good
- Good
- Fair
- Poor
- Declined

**14. If you know your primary mental health diagnosis, please tell me what it is.**

One only; no prompts

- Adjustment disorder
- Anxiety disorder
- Bipolar disorder
- Borderline personality disorder
- Other personality disorder
- Depression
- Eating disorder
- Obsessive compulsive disorder
- Perinatal depression
- PTSD
- Schizoaffective disorder
- Schizophrenia
- Other psychosis (inc drug-induced)
- Stress
- Substance misuse
- Declined
- Don't know / can't recall
- Other:__________________

**15. If you have one or more secondary mental health diagnoses, please tell me what they are.**

No prompts. Multiple responses possible.

- Adjustment disorder
- Anxiety disorder
- Bipolar disorder
- Borderline personality disorder
- Other personality disorder
- Depression
- Eating disorder
- Obsessive compulsive disorder
- Perinatal depression
- PTSD
- Schizoaffective disorder
- Schizophrenia
- Other psychosis (inc drug-induced)
- Stress
- Substance misuse
- Declined
- Don't know / can't recall
- Other:___________________

**»» The next question is about suicide and self-harm.**

**16. In the last 12 months, have you done any of the following things?**

Multiple responses possible

- Thought seriously about killing yourself
- Made a plan to kill yourself
- Attempted to kill yourself
- Deliberately done something to harm or hurt yourself, without intending to kill yourself
- None of these
- Declined

**»» Now I’d like to ask you about hospital admissions for mental health reasons.**

**17. In the last 12 months have you been admitted to hospital for mental health reasons?**

- Yes -> go to Q24
- No -> go to Q29
- Declined -> go to Q29

**18. How many times during the last 12 months were you admitted...**

If declined, use 99.

If don't know/can't recall, ask for an estimate.

If unable to estimate, use 100.

- to a mental health unit (specifically for people with mental illness)? ____________
- for mental health reasons to some other type of hospital ward? ____________
- as an involuntary mental health patient? ______________

**19. During the last 12 months, what was the total number of nights you spent in hospital for mental health reasons? It's OK if you can't remember exactly; an estimate is fine.** ___________

If declined, use 99.

**»» The next questions are about any visits you might have made to a hospital emergency department for mental health-related reasons.**

**20. During the last 12 months, have you ever been to a hospital emergency department to get help with a mental health problem?**

- Yes -> go to Q21
- No -> go to Q22
- Not sure / can't recall -> go to Q22
- Declined -> go to Q22

**21. How many times during the last 12 months did you go to a hospital emergency department for help with a mental health problem?**

**If you can't remember, please just give me an estimate.**___________

If declined, use 99.

**»» Now I’d like to ask you about your housing situation.**

**22. What kind of housing do you live in at present?**

**For example, do you live in:**

- public (social) rental housing -> go to Q24
- private rental housing -> go to Q24
- your own home, or one that you're currently buying -> go to Q24
- a boarding house -> go to Q24

**Or are you:**

- renting a room or boarding privately -> go to Q24
- couch‐surfing or in other temporary accommodation -> go to Q25
- homeless -> go to Q23
- other?-> go to Q24

**23. Please tell me how long you've been homeless.**

If the participant is moving in and out of homelessness, about how long has the current period of homelessness been? Read out response options if necessary.

- Less than 1 week -> go to Q27
- More than 1 week but less than 1 month -> go to Q27
- 1 to 2 months -> go to Q27
- 3 months or longer -> go to Q27
- Declined -> go to Q27

**24. Were there any times during the last 12 months when you were homeless or had no stable accommodation?**

- No -> go to Q26
- Yes -> go to Q28
- Declined -> go to Q26

**25. How long have you been couch surfing / had no stable accommodation?**

Estimated length of the participant's current period of housing instability

Read out response options from drop down list if necessary.

- Less than 1 week -> go to Q27
- More than 1 week but less than 1 month -> go to Q27
- 1 to 2 months -> go to Q27
- 3 months or longer -> go to Q27
- Declined -> go to Q27

**26. Who do you currently live with?**

- Living alone
- Living with partner and/or family members
- Sharing with friends/housemates
- Declined
- Other:___________________________

**27. Thinking about the last 12 months, how many times have you moved house or changed accommodation during that period?**

- None -> go to Q29
- 1 -> go to Q29
- 2 -> go to Q29
- 3 or more -> go to Q29
- Declined -> go to Q29

**28. For about how long during the last 12 months were you homeless or living in some kind of temporary accommodation?**

**If there was more than one period when you were living like that, please estimate the total length of time.**

Read out response options if necessary.

- Less than 1 week -> go to Q26
- More than 1 week, but less than 1 month -> go to Q26
- 1 to 2 months -> go to Q26
- 3 months or longer -> go to Q26
- Declined-> go to Q26

**»» The next few questions are about your employment situation.**

**29. Are you currently doing any paid work?**

For this question, 'currently' means now and over the last 2‐4 weeks.

- Yes -> go to Q30
- No -> go to Q32
- Declined -> go to Q32

**30. What sort of paid work do you do?**

See response options.

Use probing Qs to help identify whether the participant works in a 'mainstream' job for wages/salary, is self‐employed, or is paid to do work made available via:

- a 'make work' scheme (e.g., work for the dole)
- a supported employment initiative for people with specific disadvantages/disabilities/needs (e.g., mental health clubhouse programs, The Big Issue, Endeavour Foundation workshops)
- a social enterprise or similar initiative that provides employment opportunities and training/experience to people who are currently marginalised by the competitive jobs market. If unsure, use 'Other' and enter job title and/or employer's name.


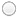
 'Real' / mainstream job for wage/salary


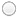
 Self-employed


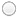
 Work for the dole or similar


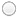
 Mental health clubhouse or similar


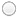
 Social enterprise or similar


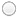
 Declined


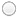
 Other: _________________________________

**31. How many hours of paid work do you normally do each week?**

**If the number of hours varies from week to week, please estimate an average number, based on the last 4 weeks.**

If declined, use 99.

_______________________

**32. Are you actively looking for any paid work at present? This includes looking for:**

- **more paid work (additional hours or an additional job)**
- **paid work with better pay or conditions**
- **a different type of paid work**
- **paid work with a different employer**
- **paid work in a different location.**
- No
- Yes, looking for paid work
- Yes, looking for MORE paid work
- Yes, looking for DIFFERENT paid work
- Yes, looking for work with BETTER PAY/CONDITIONS
- Yes, looking for paid work with a DIFFERENT EMPLOYER
- Yes, looking for paid work in a DIFFERENT LOCATION
- Declined
- Other: ______________________________

**»» Now I'd like to ask you briefly about unpaid work.**

**Unpaid work can include things like looking after children or other family members, or doing domestic work for your household (if you live with other people), as well as various kinds of voluntary work.**

**33. Are you currently doing any unpaid work?**

For this question, 'currently' means now and over the last 2 to 4 weeks.

- Yes -> go to Q34
- No -> go to Q36
- Declined -> go to Q36

**34. What type/s of unpaid work are you currently doing?**

Multiple responses possible

- Domestic work for your household
- Voluntary work in the community
- Caring for family member/s with disability, long-term illness or old age
- Looking after one or more children <15 yrs old
- Declined
- Other unpaid work: ___________________

**35. How many hours each week do you normally spend doing unpaid work?**

**If the number of hours varies from week to week, please estimate an average number, based on the last 4 weeks.**

If declined, use 99.

________________

**»» Next I have one or two questions about study.**

**36. Are you currently studying for an education or training qualification?**

For this question, 'currently studying' means enrolled at present and actively participating in learning activities (lectures, practical exercises, assessment tasks, etc), whether face‐to‐face or online.

'Currently studying' would also apply if the interview takes place during a semester break (or similar) and the participant is part-way through a study program that they will be continuing in the next semester.

- No -> go to Q38
- Yes, studying part-time -> go to Q37
- Yes, studying full-time -> go to Q37
- Declined -> go to Q38

**37. Where are you studying?**

Enter the type of study institution, if it can be identified. Use 'other' to specify either a type of institution not listed or (if the type is unknown) the name of the institution.

- TAFE or technical college
- University
- Business college
- Other: ___________________
- Declined

**»» The next questions are about mental health treatment services you've used during the last 12 months.**

**38. Other than as a hospital inpatient or at a hospital emergency department, have you had any specialised mental health treatment during the last 12 months?**

**This would be treatment/therapy you've received from a mental health professional such as a mental health nurse, psychiatrist, psychologist or psychotherapist.**

- No -> go to Q47
- Yes -> go to Q39
- Can't recall -> go to Q47
- Declined -> go to Q47

**39. Can you remember where you got that treatment, or from whom?**

**For example, did you go to any of the following?**

Read list. Multiple responses are possible, including 'Other'. However, the latter should not be required, so use further Qs to clarify this response, if selected.

*If respondent lists multiple responses, make sure to refer back to this question and follow directions to each corresponding follow-up question before moving on to the next section (Q66)***.**

- Community mental health service -> go to Q40
- Medicare-funded mental health practitioner -> go to Q41
- Other mental health practitioner (not Medicare-funded) -> go to Q42
- Don't know / can't recall -> go to Q47
- Declined -> go to Q47
- Other: ________________ -> go to Q46

**40. You said you went to a community mental health service for treatment during the last 12 months. Can you recall how many times you went there?**

**It's OK if you can't remember exactly; please just give me an approximate number.**

If declined, use 99.

______________________ -> go to Q43

**41. You mentioned that you've been to one or more Medicare-funded mental health practitioners for treatment during the last 12 months. Could you tell me how many times you did that?**

**If you can't recall exactly, an approximate number is fine.**

If declined, use 99.

______________________ -> go to Q44

**42. You said that during the last 12 months you went to one or more specialised mental health practitioners whose services were not funded through Medicare.**

**Can you recall how many times you did that?**

**An approximate number is fine if you can't remember exactly.**

If declined, use 99.

______________________ -> go to Q45

**43. Are you still going to the community mental health service?**

If participant is unsure, ask whether they have an appointment for another visit. Use 'Other' to note any remaining uncertainty.


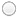
 No
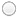
 Yes
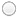
 Declined
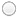
 Other: ____________ -> go to Q47

**44. Are you still going to a Medicare‐funded mental health practitioner for treatment?**

If participant is unsure, ask whether they have made another appointment. Use 'Other' to note any remaining uncertainty.


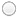
 No
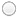
 Yes
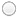
 Declined
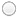
 Other: ____________ -> go to Q47

**45. Are you still seeing a mental health practitioner who isn't funded through Medicare?**

If participant is unsure, ask whether they have an appointment for another visit. Use 'Other' to note any remaining uncertainty.


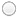
 No
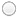
 Yes
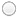
 Declined
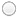
 Other: ____________ -> go to Q47

**46. You said earlier that you had been to another type of specialised mental health provider for treatment. Are you still going there for treatment?**

May need to refer back to responses to Q53.

If participant is unsure about whether they are still going to this provider, ask whether they have made another appointment. Use 'Other' to explain any further uncertainty.


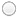
 No
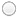
 Yes
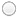
 Declined
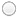
 Other: ____________ -> go to Q47

**»» The next questions are about visits to general practitioners (GPs).**

**I only want to ask about GP visits you’ve made in relation to your own health, so please ignore any times when you might have taken someone else to see a doctor.**

**47. Do you have:**

- **a regular GP**
- **a regular GP practice — a group of doctors in one location, where you usually go when you need to see a GP, or**
- **two or more GPs to whom you usually go, depending on the health issue at the time?**

'Regular' means the participant prefers/tries to go to this GP/practice, although it might not always be possible.

- No regular GP or GP practice
- Yes, regular GP
- Yes, regular GP practice
- Yes, more than one regular GP/practice
- Declined

**48. Thinking about the last 12 months, did you visit a GP — either your regular GP or another GP — for reasons related to your own health during that period?**

- No -> go to Q52
- Yes -> go to Q49
- Can't recall / Not sure -> go to Q52
- Declined -> go to Q52

**49. Can you recall the general reasons for any of your visit/s to a GP during the last 12 months?**

You don't need to give me any details, just whether your visits to a GP were for:

- Mainly physical health reasons
- Mainly mental health reasons
- Both physical and mental health reasons
- Can't recall any reasons
- Declined
- Other reasons: ____________

**50. Could you tell me how many times you've visited a GP during the last 12 months?**

**If you can't remember exactly, that's OK; please give me an approximate number.**

If declined, use 99.

_______________ -> if 99 or 0, go to Q52

**51. Could you tell me how many — or roughly how many — of those GP visits during the last 12 months were for mental health reasons, or included some discussion about your mental health?**

An example of the latter would be if the participant went to the doctor for a physical health reason, but the doctor also checked on his/her mental health.

If declined, use 99.

_______________

**»» I just have a few questions now about community services that you might be using.**

**52. Please tell me whether you are currently using any of the following types of community support services.**

For this question, 'currently' means now and over the last 2 to 4 weeks. Read the list slowly to the participant, pausing for a response to each type of service. Multiple responses are possible.

- Alcohol or other drug service -> go to Q53 and then **skip** to Q56
- Child or family support service -> go to Q53 and then **skip** to Q56
- Disability support service -> go to Q53 and then **skip** to Q56
- Domestic violence service -> go to Q53 and then **skip** to Q56
- Emergency/crisis support service -> go to Q53 and then **skip** to Q56
- Employment support service -> go to Q53 and then **skip** to Q56
- Financial counselling service -> go to Q53 and then **skip** to Q56
- Other counselling service -> go to Q53 and then **skip** to Q56
- Homelessness support service -> go to Q53 and then **skip** to Q56
- Housing service -> go to Q53 and then **skip** to Q56
- Mental health support service -> go to Q53
- Not currently using any services -> go to Q56 and end interview
- Declined to answer -> go to Q56 and end interview
- Other service: ____________ -> go to Q53 and then **skip** to Q56

| Not at all well |  | Moderately well |  | Extremely well | Declined |
| --- | --- | --- | --- | --- | --- |
| 1 | 2 | 3 | 4 | 5 | 99 |

**53. On a scale of 1 to 5, where 1 is 'not at all well' and 5 is 'extremely well', how well are these support services currently meeting your overall mental health recovery and support needs?**

**54. You've told me you're currently using a mental health support service — or possibly more than one. So just to clarify, how many mental health support services are you using at the moment?**

'At the moment' means the participant is still engaged with the service, and expects to keep using it, at least for the time being.

- 1
- 2
- 3
- More than 3
- Declined to answer

**55. And does that include the Floresco Centre?**

- No
- Yes
- Declined to answer

**56. Use this area to make any additional comments or notes on participant’s responses to one or more questions.**

__________________________________________________________________________________

__________________________________________________________________________________

__________________________________________________________________________________

__________________________________________________________________________________

__________________________________________________________________________________

__________________________________________________________________________________

__________________________________________________________________________________

__________________________________________________________________________________

__________________________________________________________________________________

__________________________________________________________________________________

**Thank you very much for answering these questions.**

**I’d just like you to do one more thing. It should only take a couple of minutes.**

- Ask participant to complete the RAS-DS.
- Give the participant a gift card as thanks.
- Make sure you get them to sign the receipt for the gift card.
- Check participant’s willingness to participate in another interview in about 6 months time.
- If yes to the above, let them know we’ll be in touch (by phone/email) to organise a date and time for the next interview.
