# Supplementary material for: A mixed methods evaluation of an integrated adult mental health service model
Source: BMC Health Serv Res. 2019 Oct 14;19:691. doi: 10.1186/s12913-019-4501-7 (PMC6791005; doi:10.1186/s12913-019-4501-7)
Supplement: Supplementary file 6 — Follow-up interview guide. (DOCX 67 kb) [file 12913_2019_4501_MOESM6_ESM.docx]

**Additional file 6: Follow-up interview guide**

| Floresco Evaluation |
| --- |
| Paper interview reference guide: Follow-up interviews  Notes:   - If there is no arrow next to an answer then go to the next question - If there is an arrow next to an answer then go to the question indicated - If the question does not say “multiple responses possible” then only one response should be recorded |

**1. Today's date _____________________**

**2. Interview number**

- Floresco 1
- Floresco 2

**As for the last interview, I'm recording only your ID code, not your name, to ensure your responses remain confidential.**

**<ID code is a combination of numbers, letters from the participant’s name and their date of birth>**

**3. Participant ID code __________­­­­­___**

**»» I'd like to start by re‐visiting a few of the demographic questions from the first interview, to see whether anything has changed since then.**

**4. What is currently your main source of income?**

For this question, 'currently' means now and over the last 2‐4 weeks.

- Salary or wages -> go to Q6
- Self-employed -> go to Q66
- Government benefits -> go to Q5
- Superannuation/investments -> go to Q6
- No income -> go to Q6
- Declined -> go to Q6
- Other: -> go to Q6

**5. What is the main type of government benefit you're currently receiving?**

i.e. the benefit that currently provides the largest amount of regular income

- ABSTUDY or AUSTUDY
- Carer payment
- Disability support pension
- Newstart
- Parenting payment
- Sickness allowance
- Special benefit
- Declined
- Other: ____________

**»» Now I have a few questions about your physical and mental health.**

**6. In general, thinking about the period since the last interview, would you describe your physical health as excellent, very good, good, fair or poor?**

- Excellent
- Very good
- Good
- Fair
- Poor
- Declined

**7. In general, thinking about the period since the last interview, would you describe your mental health as excellent, very good, good, fair or poor?**

- Excellent
- Very good
- Good
- Fair
- Poor
- Declined

**»» The next question is about suicide and self-harm.**

**8. Since the last interview, have you done any of the following things?**

Multiple responses possible

- Thought seriously about killing yourself
- Made a plan to kill yourself
- Attempted to kill yourself
- Deliberately done something to harm or hurt yourself, without intending to kill yourself
- None of these
- Declined

**»» Now I’d like to ask you about hospital admissions for mental health reasons.**

**9. Since the last interview, have you been admitted to hospital for mental health reasons?**

- Yes -> go to Q10
- No -> go to Q12
- Declined-> go to Q12

**10. During that period, how many times were you admitted...**

If declined, use 99.

If don't know/can't recall, ask for an estimate.

If unable to estimate, use 100.

to a mental health unit (specifically for people with mental illness)? ____________

to some other type of hospital ward? ____________

as an involuntary patient? ______________

**11. During that period, what was the total number of nights you spent in hospital for mental health reasons? It's OK if you can't remember exactly; an estimate is fine.**

If declined, use 99.

_______________

**»» The next questions are about any visits you might have made to a hospital emergency department for mental health-related reasons**

**12. During the period since the last interview, have you been to a hospital emergency department to get help with a mental health problem?**

- Yes -> go to Q13
- No -> go to Q14
- Not sure/can't recall -> go to Q14
- Declined-> go to Q14

**13. How many times during that period did you go to a hospital emergency department for help with a mental health problem?**

If you can't remember, please just give me an estimate.

If declined, use 99.

_____________________

**»» Now I’d like to ask you about your housing situation.**

**14. What kind of housing do you live in at present?**

For example, do you live in:

- public (social) rental housing -> go to Q17
- private rental housing -> go to Q17
- your own home, or one that you're currently buying -> go to Q17
- a boarding house -> go to Q17

Or are you:

- renting a room or boarding privately -> go to Q17
- couch‐surfing or in other temporary accommodation -> go to Q16
- homeless -> go to Q15
- other?-> go to Q17

**15. Please tell me how long you've been homeless.**

If the participant is moving in and out of homelessness, about how long has the current period of homelessness been? Read out response options if necessary.

- Less than 1 week -> go to Q18
- More than 1 week but less than 1 month -> go to Q18
- 1 to 2 months -> go to Q18
- 3 months or longer -> go to Q18
- Declined -> go to Q18

**16. How long have you been couch surfing / had no stable accommodation?**

Estimated length of the participant's current period of housing instability

Read out response options from drop down list if necessary.

- Less than 1 week -> go to Q18
- More than 1 week but less than 1 month -> go to Q18
- 1 to 2 months -> go to Q18
- 3 months or longer -> go to Q18
- Declined -> go to Q18

**17. Who do you currently live with?**

- Living alone -> go to Q19
- Living with partner and/or family members -> go to Q19
- Sharing with friends/housemates -> go to Q19
- Declined -> go to Q19
- Other: ___________________________ -> go to Q19

**18. Thinking about the period since the last interview, how many times have you moved house or changed accommodation during that period?**

- None -> go to Q21
- 1 -> go to Q21
- 2 -> go to Q21
- 3 or more -> go to Q21
- Declined -> go to Q21

**19. Were there any times since the last interview, when you were homeless or had no stable accommodation?**

- No -> go to Q18
- Yes -> go to Q20
- Declined -> go to Q18

**20. For about how long since the last interview were you homeless or living in some kind of temporary accommodation?**

If there was more than one period when you were living like that, please estimate the total length of time. Read out response options if necessary.

- Less than 1 week -> go to Q18
- More than 1 week, but less than 1 -> go to Q18
- 1 to 2 months -> go to Q18
- 3 months or longer -> go to Q18
- Declined -> go to Q18

**»» The next few questions are about your employment situation.**

**21. Are you currently doing any paid work?**

For this question, 'currently' means now and over the last 2‐4 weeks.

- Yes -> go to Q22
- No -> go to Q24
- Declined -> go to Q24

**22. What sort of paid work do you do?**

See response options.

Use probing Qs to help identify whether the participant works in a 'mainstream' job for wages/salary, is self‐employed, or is paid to do work made available via:

- a 'make work' scheme (e.g., work for the dole)
- a supported employment initiative for people with specific disadvantages/disabilities/needs (e.g., mental health clubhouse programs, The Big Issue, Endeavour Foundation workshops)
- a social enterprise or similar initiative that provides employment opportunities and training/experience to people who are currently marginalised by the competitive jobs market. If unsure, use 'Other' and enter job title and/or employer's name.
- 'Real' / mainstream job for wage/salary
- Self-employed
- Work for the dole or similar
- Mental health clubhouse or similar
- Social enterprise or similar
- Declined
- Other:__________

**23. How many hours of paid work do you normally do each week?**

If the number of hours varies from week to week, please estimate an average number, based on the last 4 weeks.

If declined, use 99.

_______________________

**24. Are you actively looking for any paid work at present?**

This includes looking for:

- more paid work (additional hours or an additional job)
- paid work with better pay or conditions
- a different type of paid work
- paid work with a different employer
- paid work in a different location.
- No
- Yes, looking for paid work
- Yes, looking for MORE paid work
- Yes, looking for DIFFERENT paid work
- Yes, looking for work with BETTER PAY/CONDITIONS
- Yes, looking for paid work with a DIFFERENT EMPLOYER
- Yes, looking for paid work in a DIFFERENT LOCATION
- Declined
- Other: ________________

**»» Now I'd like to ask you briefly about unpaid work.**

**Unpaid work can include things like looking after children or other family members, or doing domestic work for your household (if you live with other people), as well as various kinds of voluntary work.**

**25. Are you currently doing any unpaid work?**

For this question, 'currently' means now and over the last 2 to 4 weeks.

- Yes -> go to Q26
- No -> go to Q28
- Declined -> go to Q28

**26. What type/s of unpaid work are you currently doing?**

Multiple responses possible

- Domestic work for your household
- Voluntary work in the community
- Caring for family member/s with disability, long-term illness or old age
- Looking after one or more children <15 yrs old
- Declined
- Other unpaid work:

**27. How many hours each week do you normally spend doing unpaid work?**

If the number of hours varies from week to week, please estimate an average number, based on the last 4 weeks. If declined, use 99.

________________

**»» Next I have one or two questions about study.**

**28. Are you currently studying for an education or training qualification?**

For this question, 'currently studying' means enrolled at present and actively participating in learning activities (lectures, practical exercises, assessment tasks, etc), whether face‐to‐face or online.

'Currently studying' would also apply if the interview takes place during a semester break (or similar) and the participant is part-way through a study program that they will be continuing in the next semester.

- No -> go to Q30
- Yes, studying part-time -> go to Q29
- Yes, studying full-time -> go to Q29
- Declined -> go to Q30

**29. Where are you studying?**

Enter the type of study institution, if it can be identified.

Use 'other' to specify either a type of institution not listed or (if the type is unknown) the name of the institution.

- TAFE or technical college
- University
- Business college
- Other: ___________________
- Declined

**»» The next questions are about mental health treatment services you've used since the last interview.**

**30. Other than as a hospital inpatient or at a hospital emergency department, have you had any specialised mental health treatment since the last interview?**

This would be treatment/therapy you've received from a mental health professional such as a mental health nurse, psychiatrist, psychologist or psychotherapist.

- No -> go to Q39
- Yes -> go to Q31
- Can't recall -> go to Q39
- Declined -> go to Q39

**31. Can you remember where you got that treatment, or from whom?**

For example, did you go to any of the following? Read list, multiple responses possible, including 'Other'. However, the latter should not be required, so use further Qs to clarify this response, if selected.

**If respondent lists multiple responses, make sure to refer back to this question and follow directions to each corresponding follow up question before moving on to the next section (Q39).**

- Community mental health service -> go to Q32
- Medicare-funded mental health practitioner -> go to Q33
- Other mental health practitioner (not Medicare-funded) -> go to Q34
- Don't know / can't recall-> go to Q39
- Declined-> go to Q39
- Other: _______________-> go to Q38

**32. You said you went to a community mental health service for treatment since the last interview.**

Can you recall how many times you went there? It's OK if you can't remember exactly; please just give me an approximate number. If declined, use 99.

___________________-> go to Q35

**33. You mentioned that you've been to one or more Medicare-funded mental health practitioners for treatment since the last interview.**

Could you tell me how many times you did that? If you can't recall exactly, an approximate number is fine. If declined, use 99.

___________________-> go to Q36

**34. You said that since the last interview you went to one or more specialised mental health practitioners whose services were not funded through Medicare.**

Can you recall how many times you did that? An approximate number is fine if you can't remember exactly. If declined, use 99.

___________________-> go to Q37

**35. Are you still going to the community mental health service?**

If participant is unsure, ask whether they have an appointment for another visit. Use 'Other' to note any remaining uncertainty.


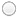
 No
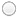
 Yes
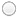
 Declined
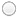
 Other: ____________ -> go to Q39

**36. Are you still going to a Medicare‐funded mental health practitioner for treatment?**

If participant is unsure, ask whether they have made another appointment. Use 'Other' to note any remaining uncertainty.


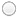
 No
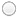
 Yes
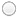
 Declined
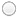
 Other: ____________ -> go to Q39

**37. Are you still seeing a mental health practitioner who isn't funded through Medicare?**

If participant is unsure, ask whether they have an appointment for another visit. Use 'Other' to note any remaining uncertainty.


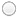
 No
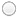
 Yes
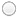
 Declined
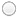
 Other: ____________ -> go to Q39

**38. You said earlier that you had been to another type of specialised mental health provider for treatment. Are you still going there for treatment?**

May need to refer back to responses to Q31.

If participant is unsure about whether they are still going to this provider, ask whether they have made another appointment. Use 'Other' to explain any further uncertainty.


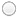
 No
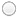
 Yes
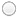
 Declined
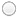
 Other: ____________

**»» The next questions are about visits to general practitioners (GPs)**

**I only want to ask about GP visits you’ve made in relation to your own health, so please ignore any times when you might have taken someone else to see a doctor.**

**39. You’ve answered this question before, but I’d like to check on any changes since then. So please tell me whether you have:**

- **a regular GP**
- **a regular GP practice ‐‐ a group of doctors in one location, where you usually go when you need to see a GP, or**
- **two or more GPs to whom you usually go, depending on the health issue at the time?**

'Regular' means the participant prefers/tries to go to this GP/practice, although it might not always be possible.

- No regular GP or GP practice
- Yes, regular GP
- Yes, regular GP practice
- Yes, more than one regular GP/practice
- Declined

**40. Thinking about the period since the last interview, did you visit a GP ‐ either your regular GP or another GP ‐ for reasons related to your own health during that period?**

- No -> go to Q44
- Yes -> go to Q41
- Can't recall/ Not sure -> go to Q44
- Declined -> go to Q44

**41. Can you recall the general reasons for any of your visit/s to a GP since the last interview?**

**You don't need to give me any details, just whether your visits to a GP were for:**

- Mainly physical health reasons
- Mainly mental health reasons
- Both physical and mental health reasons
- Can't recall any reasons
- Declined
- Other reasons: ____________

**42. Could you tell me how many times you've visited a GP since the last interview?**

If you can't remember exactly, that's OK; please give me an approximate number.

If declined, use 99.

_______________ -> if 99 or 0 go to Q44

**43. Could you tell me how many ‐‐ or roughly how many ‐‐ of those GP visits since the last interview were for mental health reasons, or included some discussion about your mental health?**

An example of the latter would be if the participant went to the doctor for a physical health reason, but the doctor also checked on his/her mental health. If declined, use 99.

_______________

**»»I just have a few questions now about community services you might have used since the last interview.**

**44. Please tell me whether you’ve used any of the following types of community support services at any time since the last interview.**

Read the list slowly to the participant, pausing for a response to each type of service. Multiple responses are possible. The question covers the whole period since the last interview (including services that the participant has used during that time, but may not still be using.

- Alcohol or other drug service
- Child or family support service
- Disability support service
- Domestic violence service
- Emergency/crisis support service
- Employment support service
- Financial counselling service
- Other counselling service
- Homelessness support service
- Housing service
- Mental health support service
- Not currently using any services -> go to Q50
- Declined to answer -> go to Q50
- Other service: ____________

**45. And which of these support services are you still using at the moment?**

The question is about the support services the participant has used since the last interview AND is still engaged with at the time of this interview. Read the list slowly, pausing for a response to each service type, and skip those types that you’re confident the participant hasn’t used since the last interview (as indicated by the previous question). Multiple responses are possible.

- Alcohol or other drug service -> do Q46 and then **skip** to Q50
- Child or family support service -> do Q46 and then **skip** to Q50
- Disability support service -> do Q46 and then **skip** to Q50
- Domestic violence service -> do Q46 and then **skip** to Q50
- Emergency/crisis support service -> do Q46 and then **skip** to Q50
- Employment support service -> do Q46 and then **skip** to Q50
- Financial counselling service -> do Q46 and then **skip** to Q50
- Other counselling service -> do Q46 and then **skip** to Q50
- Homelessness support service -> do Q46 and then **skip** to Q50
- Housing service -> do Q46 and then **skip** to Q50
- Mental health support service -> go to Q46 onwards (complete all questions in this section)
- Not currently using any services -> go to Q50
- Declined to answer -> go to Q50
- Other service: ____________ -> do Q46 and then **skip** to Q50

**46. On a scale of 1 to 5, where 1 is 'not at all well' and 5 is 'extremely well', how well are these support services currently meeting your overall mental health recovery and support needs?**

| Not at all |  | Moderately |  | Extremely |  |
| --- | --- | --- | --- | --- | --- |
| well |  | well |  | well |  |
| 1 | 2 | 3 | 4 | 5 |  |

Declined

**47. You've told me you're currently using a mental health support service ‐ or possibly more than one. So just to clarify, how many mental health support services are you using at the moment?**

'At the moment' means the participant is still engaged with the service, and expects to keep using it, at least for the time being.

- 1
- 2
- 3
- More than 3
- Declined to answer

**48. And does that include the Floresco Centre?**

- No
- Yes
- Declined to answer

**49. Could you please tell me, just briefly, why you’re not still using the Floresco Centre for mental health support?**

The reason may be a positive one, but if participants seem reluctant to answer, remind them that their responses will remain confidential and will not be able to be linked to them personally. Use 99 if participant declines to answer.

__________________________________________________________________________________

__________________________________________________________________________________

**50. Are there any issues that are currently affecting your mental health recovery in a negative way, and that you're not getting some kind of support to deal with?**

This might be support from health or community services, or from family or friends, or perhaps a combination of these.

- No -> go to Q53
- Yes -> go to Q51
- Not sure -> go to Q53
- Declined -> go to Q53

**51. Could you please tell me, just briefly, what kind/s of issues these are?**

**You don't need to tell me the details; just a general description is fine.**

**For example, are you referring to a housing or employment issue, or perhaps a relationship issue?**

__________________________________________________________________________________

__________________________________________________________________________________

__________________________________________________________________________________

**52. Please tell me the reason/s why are you not getting any kind of help to deal with this/these issues.**

Try to avoid prompting. You might need to listen to the whole answer and then fit it into one or more of the options. Multiple responses are possible.

- Can manage OK on my own
- On a waiting list for support services
- Haven't tried to get any help
- Don't know how/where to get the right kind of help
- Tried to get support, but not eligible
- No appropriate support services available
- Support services are too difficult to access
- No family
- No family able and/or willing to help
- No friends
- No friends able and/or willing to help
- Declined to answer
- Other reason

**53. Use this area to make any additional comments or notes on participant’s responses to one or more questions**

__________________________________________________________________________________

__________________________________________________________________________________

__________________________________________________________________________________

__________________________________________________________________________________

__________________________________________________________________________________

__________________________________________________________________________________

__________________________________________________________________________________

__________________________________________________________________________________

__________________________________________________________________________________

__________________________________________________________________________________

**Thank you very much for answering these questions.**

**I’d just like you to do one more thing. It should only take a couple of minutes.**

- Ask participant to complete the RAS-DS
- Give the participant a gift card as thanks
- Make sure you get them to sign the receipt for the gift card.
