# Supplementary material for: A mixed methods evaluation of an integrated adult mental health service model
Source: BMC Health Serv Res. 2019 Oct 14;19:691. doi: 10.1186/s12913-019-4501-7 (PMC6791005; doi:10.1186/s12913-019-4501-7)
Supplement: Supplementary file 7 — Examples of stakeholder comments on barriers to service integration at Floresco. (DOCX 39 kb) [file 12913_2019_4501_MOESM7_ESM.docx]

**Additional file 7: Examples of stakeholder comments on barriers to service integration at Floresco**

| **Barriers/challenges** | **Examples of stakeholder comments** |
| --- | --- |
| Bringing staff from four different NGOs together to work as one team | *Organisations don't want to lose their identity and their sense of their expertise* (Floresco staff member).  *It was a big challenge, I think, to try and put together something that accommodated good practice from a range of perspectives* (Floresco staff member, regarding development of Floresco’s practice manual).  *Organisations like to manage their organisational risk … What's going to go wrong and how can I control this? The only way you can control it is to separate and silo the services which we don’t want to do* (Aftercare manager).  *Just an example of how we had to navigate through the partners … because for* [Aftercare] *and some of the other partners, that's very important to them* [but] *we don't do duress alarms … so for little things like that it's been a real negotiation between the partners* (NGO partner representative)*.*  [One NGO partner was] s*trongly against our clinical team leader and our service manager providing supervision to* [its] *staff. They also wanted staff to report* [crises] *directly to their own managers or supervisors… It was slowing the process down and delaying those crisis response procedures* (Floresco staff member).  *I was very confused for a long time as to who I was working for* (Floresco support worker). |
| Barriers to integrating with the MHS | *It's the NGO sector attempting to pull the levers, which they don't — the sector doesn't have that power and influence* (Aftercare manager).  *The major challenges have been … around the freedom that* [the MHS] *has to participate in these types of models. They've got a whole range of restrictions ... The leadership was on side, but their system barriers … that's been one of the major, a major sort of challenge, because you need* [the MHS] *at the table* (Aftercare manager).  *The integration is in two parts. One, you've got the integration with the* [MHS] … *Then you've got the integration of NGOs* (NGO partner representative). |
| Barriers to systematic information sharing | *Shared information is major. Different organisations recording information on different systems. At one point* [they] *were hoping that our staff would do a double — like a duplication. That's just not going to happen, it was just too difficult* (MHS staff member).  [The MHS] *really were the only ones that had the major problem around the one client information management system … all of the difficulties around confidentiality* … (Aftercare manager).  *We still haven’t overcome that, the system barrier* (Floresco staff member).  *Where we've fallen down is with the telling of the story over and over again. It's still sometimes necessary because we don't share databases* (Floresco staff member).  *We had all the intent that it was going to be an integrated record, but the problem has been … allocating resources to make sure it's being used correctly. People … just pumped in whatever they thought needed to be done but no one actually knew the implications of what they were entering and how that looked* [in terms of extracting and reporting] *data and activity* (Aftercare manager). |
| Resourcing challenges | *The funding and the resources behind it needed to be more intense. For example, I think the funding for a clinical team leader, that person — it wasn't sufficient funding* (Floresco staff member).  *I think one of the biggest challenges … is the complexity of the clients that are coming to Floresco* [is] *much, much higher than what we originally thought. … What they're needing Floresco to provide to them, where there are gaps in the community, is more than what we thought* (Aftercare manager).  *We are not getting enough funding to justify, say, employing social workers, or people with experience … Some of the best staff we’ve had haven’t had those qualifications, but the salaries that they’re on aren’t enough to justify the work they’re doing. With or without qualifications* (Floresco staff member).  *It needs more funding to be able to do what it does... The* [MHS] *needs in some way to be supported to participate… The biggest problem around the private system, engaging them, is that — particularly GPs, but even some allied health professionals — do not just want to work under MBS, they want to charge a* [fee] (Aftercare manager).  *I would love to have two clinicians to send down* [to Floresco] *every day but I don't have the resources* (MHS staff member). |
| Staffing problems | *We've gone through two service managers.* [X is] *probably the third team leader. I can't even count how many support workers we've gone through* (Floresco staff member).  *It was not the role that I expected to walk into. I had a completely different vision of what was going to be expected of me. … We are not support workers, we are case managers* (Floresco support worker).  *Looking generally at it, most cases it’s workload... Many, many things that are so emotionally draining and such high pressure work... So they’re currently funded as support workers. But in my mind, they’re doing short-term case management... So the money isn’t worth the pressure* (Floresco staff member).  *We have been on many occasions understaffed for a long time because the* [partner] *organisations don’t see the pressure that we’re under here. … So they don’t understand the urgency of recruiting when a position is vacant* (Floresco staff member).  *There's been challenges generally recruiting staff to the service, so management as well as support staff, who really understand … that integrated model.... Then that has had an impact on how we work with our partners, and how we recruit other staff* (Aftercare manager).  *There was quite a marked difference in the approach of the two service managers … Before, it appeared … that there was a close eye on how the model was progressing. I don't know that we could say that there is that at the moment* (NGO partner representative).  *Recruiting the right people to do the job, whether they were working for us as non-government organisations, or getting private practitioners, such as particularly GPs* [was] *like pulling teeth* (Aftercare manager). |
| Recruiting and retaining GPs and private mental health practitioners | *We haven't been able to recruit private practitioners to the level that we thought we would, which has then impacted the amount of clinical support that is provided* (Aftercare manager)*.*  *They're not getting private practitioners because a private practitioner probably would make more money on their own than actually working under a Floresco model* (private practitioner).  *GPs and private practitioners, you know, why come to this particular location, why work in this particular context?* (Aftercare manager).  *I don't think that there's been the supports in place. I pay for all of my own clinical supervision* (private practitioner). |
| Responding to demand | *I think that we were all blown away when* [Floresco] *opened the doors and … just got absolutely inundated* (MHS staff member).  *The idea of a one-stop shop is brilliant, but you've got to be able to adequately resource that … there's fairly long waiting lists, and that's difficult, because people still are referred every day, and usually when they're referred there's crucial issues going on in their lives that need to be responded to in a timely manner. … The backlog is becoming bigger, and staff are feeling pressured* (private practitioner). |
| Responding to clinical need | *Originally, we had very much set it up as a support worker kind of model, and what we're seeing now … is we need to balance that support work model with a fair bit of clinical support, probably more than we initially thought. Although we always thought it would have a clinical component, I think that balance isn't there as it should be* (Aftercare manager).  *The idea was for it to be a bit of a one-stop shop but then we're finding, because the waitlist* [for private practitioners] *is so long, we're referring out for counselling support or other things just because we don't have the capacity to be able to offer that at the time* (Floresco staff member).  *We're needing to look at, okay, how else do we bring in that clinical support if it's not going to be through private practitioners, because it's needed* (Aftercare manager). |
| Operating as a consortium | *We have partner organisations offsite who recruit people without necessarily taking into consideration the workplace culture here and then those staff kind of come over here and then there has been issues, just in terms of workplace culture, office morale, that sort of thing, because of recruitment decisions that* [the partner] *organisations don't necessarily have to* [deal with] (Floresco support worker).  *It served a purpose at the very start for all of the organisations to be involved, and to have buy-in to the service, but I'm not sure that it's the best way… There's been some real positives with it, but I think there are some very real challenges* (Aftercare manager).  *It's not the most effective use of money, for any of us. I think that possibly if you brought that money in and then had in-kind co-located services rather than subcontracted co-located services, that would be a better way to be managing that* (Aftercare manager).  *From a clinical governance perspective … we still have some real challenges* (Aftercare manager).  *It's overly complex in terms of having a range of mental health NGOs who do similar sorts of things, and have similar sorts of capabilities. … One agency probably could have done the work of at least two of them, and you might have had partnership with one of them, maybe* (Aftercare manager).  *Look, it can work, it's worked, but the pressure it puts on the management structure … I think it's a big ask* (Aftercare manager).  *As consortium partners we probably haven't helped to achieve service integration beyond our own agency being a strong participant… It was definitely about ‘are we achieving our outcomes?’… Our energies went probably into that rather than into the bigger picture model* (NGO partner representative).  *We are in a different climate now than when the Floresco model started. Because we've got — there are huge pressures on NGOs to find their way in NDIS land. That could have a bigger effect on the Floresco model than anything else… So we've had the big push — collaborate, collaborate, collaborate. Then it's like, oh hello. Well, you all need to vie for … you know, you'll need to fight for your funding* (NGO partner representative). |
| Inconsistent leadership and governance | *Of course, having lost* [X], *who was a big, big motivator…* [and] *had a way of getting things done… It shouldn't rely on one person.* (MHS staff member).  *Executive endorsement and leadership and drive down to keep this happening has been lacking.*.*. I don’t think that the management of this — from a senior management point of view's gone very well. There should have been much more commitment, much more energy given to the model and what we were trying to achieve.* (Aftercare manager).  *At the start … there was a really energetic, cohesive group and we were always there and we knew* [what we were going to do]*. Then I think there's a couple of organisations who drip different people in and out all the time and you just haven't got that consistency.* (Governance Committee member).  *There's varying levels of seniority at that steering committee as well.* (Governance Committee member).  *Service integration governance is — I think that is something that is still quite new as well. How do you, you know, it's not a parcel or a piece of work that's easy to find to kind of piggyback off* (NGO partner representative).  *I think we should have been thinking about things like permanency of funding and the direction. I don't know that we got all of that in the steering committee* (Governance Committee member). |
